# Simulation-Based Two-Stage Scheduling Optimization Method for Carrier-Based Aircraft Launch and Departure Operations

**DOI:** 10.3390/e27070662

**Published:** 2025-06-20

**Authors:** Jue Liu, Nengjian Wang

**Affiliations:** College of Mechanical and Electrical Engineering, Harbin Engineering University, No. 145 Nantong Street, Nangang District, Harbin 150001, China; wangnengjian@hrbeu.edu.cn

**Keywords:** simulation, carrier-based aircraft, scheduling optimization, algorithm improvement, departure operation

## Abstract

The scheduling of carrier-based aircraft departure operations is subject to stringent temporal, spatial, and resource constraints. Conventional approaches struggle to yield exact solutions or provide a comprehensive mathematical description of this complex, dynamic process. This study proposes a simulation-based optimization method, establishing a high-fidelity simulation model for aircraft departure scheduling. To address the coupled challenges of path planning under spatial constraints and station matching/sequencing under operational constraints, we developed (1) a deep reinforcement learning (DRL)-based path planning algorithm (AAE-SAC), and (2) an enhanced particle swarm optimization (PSO) algorithm (LTA-HPSO). This integrated two-stage framework, termed LTA-HPSO + AAE-SAC, facilitates efficient, collision-free departure scheduling optimization. Simulation experiments across varying sortie scales were conducted to validate the framework’s effectiveness and robustness. Notably, for a complex scenario involving 24 aircraft with diverse priorities and stringent spatial constraints, LTA-HPSO + AAE-SAC achieved an average solution time of 185.19 s, reducing scheduling time by 26.18% and 49.54% compared to benchmark algorithms (PSO + Heuristic and PSO + SAC, respectively). The proposed LTA-HPSO + AAE-SAC framework significantly enhances the quality and robustness of carrier-based aircraft departure scheduling.

## 1. Introduction

Aircraft carriers are critical combat assets, and the safe and efficient execution of aircraft launch and recovery operations directly determines sustained strike capability. Enhancing launch and recovery efficiency has, thus, remained a focal point in carrier-based aircraft support scheduling research. Carrier-based aircraft operations have a significantly different launch phase compared with land-based aircraft operations [1,2]. This can be attributed to (1) increased scheduling complexity due to confined deck space; (2) tighter departure sequencing due to limited takeoff runway length and quantity; and (3) takeoff challenges due to dynamic deck motion during maritime operations. Hence, the development of rational departure scheduling plans to minimize the total launch time, reduce resource occupation duration, and maintain safe, orderly, and efficient deck operations is paramount for optimizing the departure scheduling process of shipboard aircraft.

Existing research on carrier-based aircraft scheduling has predominantly addressed full-process launch and recovery operations. Early studies, such as Jewell et al. [3], analyzed the impact of support operations on operational capability. Subsequent initiatives aimed to reduce planning time through decision support systems [4,5,6]. A significant milestone was the development of MIT’s Deck Operations Course of Action Planner (DCAP) [7], integrating autonomous scheduling algorithms with simulation validation to enable human-in-the-loop decision-making. Recent research has increasingly leveraged intelligent algorithms and simulation technologies. Ghosh Dastidar and Frazzoli [8] optimized support operations using queuing networks and differential evolution, considering nominal failure rates. Michini and How [9] formulated a Markov decision process incorporating inverse reinforcement learning for optimal scheduling policies, albeit facing scalability challenges with larger fleets. Zhang et al. [6] developed a simulation-based deck operation planning system incorporating human–computer interaction. Yuan et al. [10] modeled deck operations as an integer programming problem with hybrid periodic/event-driven scheduling, solved via a dual-population genetic algorithm, and demonstrated its effectiveness in dynamic environments.

However, dedicated research specifically focusing on the departure sub-process remains relatively limited. Wan et al. [11] abstracted the departure scheduling problem as a hybrid flow-shop model with blocking constraints, solved using cross-entropy algorithms and heuristic rules. Liu et al. [12] approached the same problem as a flexible job-shop scheduling problem, employing mixed-integer programming and a hybrid genetic-simulated annealing algorithm with rescheduling strategies. While these studies advance launch capability optimization, key limitations persist: (1) insufficient granularity in modeling the departure sub-process; (2) inadequate incorporation of critical spatial constraints, such as path interference and wake turbulence; and (3) premature convergence and susceptibility to local optima in single-model, single-algorithm approaches.

To address these gaps, this research focuses on optimizing the scheduling problem for carrier-based aircraft departure operations, explicitly emphasizing the unique characteristics of the constrained deck operation space. We adopt a simulation-based optimization approach, establishing both a high-fidelity simulation model and an associated mathematical programming model for aircraft departure operations. Crucially, the simulation model captures, in real-time, critical factors like path conflicts, wake interference, and multi-aircraft coordination—factors challenging to observe in conventional numerical simulations yet critically impacting operational capacity. Specifically, we developed a two-stage optimization framework: Stage 1 employs a deep reinforcement learning (DRL)-based path planning algorithm (AAE-SAC) to solve the spatial path optimization problem under dynamic constraints; Stage 2 utilizes an enhanced particle swarm optimization (PSO) algorithm (LTA-HPSO) to solve the resource allocation (station selection) and sequencing (departure order) problem. This integrated two-stage framework is termed LTA-HPSO + AAE-SAC. Comprehensive simulation experiments and comparative performance analysis were conducted to validate the feasibility and superiority of the proposed LTA-HPSO + AAE-SAC framework for carrier-based aircraft departure scheduling. While two-stage optimization has demonstrated feasibility in other scheduling domains [13], our work represents its first application to the complex problem of coordinated multi-aircraft departure scheduling within the highly constrained deck environment of an aircraft carrier.

## 2. Problem Description and Modeling

### 2.1. Problem Description

**Figure 1:**
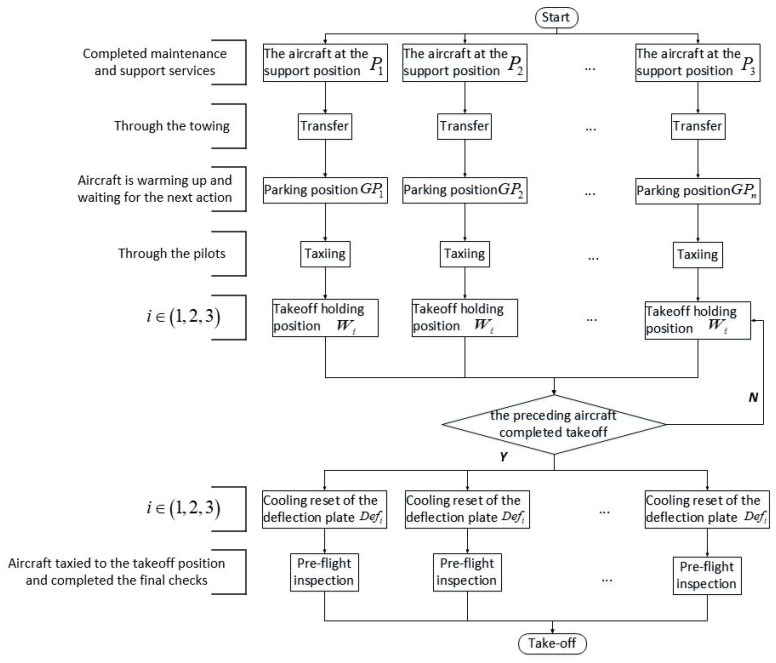
Operational flowchart for carrier-based aircraft departure.

In naval carrier operations, carrier-based aircrafts typically execute missions in continuous, high-intensity, or mixed-task sortie patterns. Prior to mission execution, aircraft undergo maintenance, logistical support, and armament loading at designated support positions. To prevent interference with subsequent operations, serviced aircraft are relocated to assigned parking positions. Subsequently, under deck officers’ commands, they taxi to designated launch positions to await takeoff authorization [14]. The departure workflow comprises five operational phases (Figure 1):

#### 2.1.1. Aircraft–Position Matching

Upon mission initiation, a predefined number of aircraft must depart to reach designated airspace. The required number of aircraft is determined by operational demands. When the number of serviced aircraft (post maintenance and logistical support) meets mission requirements, they are allocated to preassigned parking positions to await departure orders. If deck resources are insufficient, additional aircraft are transported from hangars to assigned parking positions.

#### 2.1.2. Aircraft Transfer and Taxiing

Post-service, aircrafts are towed by tractors to parking positions. During standby, engines are activated for warm-up. Upon receiving departure instructions, pilots taxi the aircraft to predetermined launch-ready positions.

#### 2.1.3. Jet Blast Deflector (JBD) Cooling and Reset

The JBD redirects engine exhaust during takeoff to mitigate deck interference, enhancing operational efficiency. Post-launch, the JBD undergoes mandatory cooling (requiring several minutes) to dissipate thermal loads before resetting. Only after JBD reset can subsequent aircraft advance from launch-ready positions to the catapult-assisted runway.

#### 2.1.4. Preflight Inspection

Prior to launch, deck crews verify aircraft status, JBD alignment, catapult integrity, runway conditions, and pilot readiness.

#### 2.1.5. Takeoff and Departure

Post-verification, pilots and launch officers execute departure upon deck commander’s command. Aircraft are then guided by launch officers to initiate catapult-assisted takeoff, completing the departure sequence.

### 2.2. Notation

- Indices

i,j: Number of aircraft in the carrier, i=1,…,n, j=1,…,n, i≠j;

- Sets

P_C: Set of all coordinate points within the 2D deck plane, P_C=Lp∪Mp∪Rp;

Lp: Coordinate set of the deck port-side area, Lp∈P_C;

Mp: Coordinate set of the deck landing area, Mp∈P_C;

Rp: Coordinate set of the deck starboard area, Rp∈P_C;

P_CF: Set of points of solid obstacles in the 2D deck plane, P_CF∈P_C;

$P_{i}$, $P_{j}$: Set of geometric center points of the outer contours of aircraft i and j;

$Angle\_P_{i}$, $Angle\_P_{j}$: Angle sets of the encountered aircraft i and j in the deck coordinate system;

$P_{Fj}$: Coordinate of the geometric center point of the j-th obstacle;

- Parameter

$v_{i}\;,v_{j}$: Current traveling speeds of the encountering aircraft i and j;

$\Delta v_{i}\;,\Delta v_{j}$: Speed increment of aircraft i and j;

$\Delta \theta_{i}\;,\Delta \theta_{j}$: Increment of the turning angle of aircraft i and j;

$\Delta \theta_{i\max},\;\Delta \theta_{j\max}$: Maximum turning angles of aircraft i and j;

$l_{F}$: Geometric length of aircraft within the 2D plane of the deck;

$l_{C}$: Geometric length of the tractor in the 2D plane of the deck;

$l_{W}$: Maximum geometric length of the wake generated by the aircraft during taxiing in a 2D plane;

$d_{S\min}$: Minimum safe distance before aircraft collision;

$d_{F\min}$: Minimum safe distance between the outer contours of aircraft and obstacles;

$d_{SLi}$: Distance between the aircraft i and the outer contour of the deck port-side area;

$d_{FLi}$: Distance between the aircraft i and the obstacles in the port-side area;

$d_{SRi}$: Distance between the aircraft i and the outer contour of the deck starboard area;

$d_{FRi}$: Distance between the aircraft i and the obstacles in the starboard area;

$d_{FMi}$: Distance between the aircraft i and the obstacles in the landing area;

${{\mathrm{Pr}}_{i}}^{O}$: Initial priority of aircraft i departure operations;

${\mathrm{Pr}}_{Ci}$: Priority value of the type of aircraft i;

${\mathrm{Pr}}_{Mi}$: Priority value of the mission type of aircraft i;

${\mathrm{Pr}}_{Si}$: Priority value of the total travel distance of aircraft i;

${\mathrm{Pr}}_{Wi}$: Waiting time priority value for the departure operation of aircraft i;

${\mathrm{Pr}}_{SRi}$: Priority value of the remaining distance ratio of the aircraft i;

$S_{i},S_{j}$: Total length of the travel paths of aircraft i and i;

$T_{Wi},T_{Wj}$: Already waiting time for the transfer process of aircraft i and i;

$S_{Ci},S_{Cj}$: Remaining distance of aircraft i and j at the current moment;

- Decision variables

$d_{S}$: Safe distances for aircraft collision detection;

${\mathrm{Pr}}_{i}$: Dynamic priority of aircraft i at the current moment;

$S_{Ri},\;S_{Rj}$: Ratio of the remaining distances of aircraft i and j at the current moment;

$C_{i}$: Whether conflicts occur during the scheduling of aircraft i or not;

$T_{\max}$: The maximum scheduling time for aircraft depature operation;

$x_{i,Pp}$: Whether aircraft i occupy the parking position (Pp) or not;

### 2.3. Simulation Model Development for Carrier-Based Aircraft Departure Operations

We developed a discrete-event simulation platform using C + + and O2DES framework [15] to model spatial deck constraints. The main components of the platform are as follows:

#### 2.3.1. Environmental Resource Modeling

**Figure 2:**
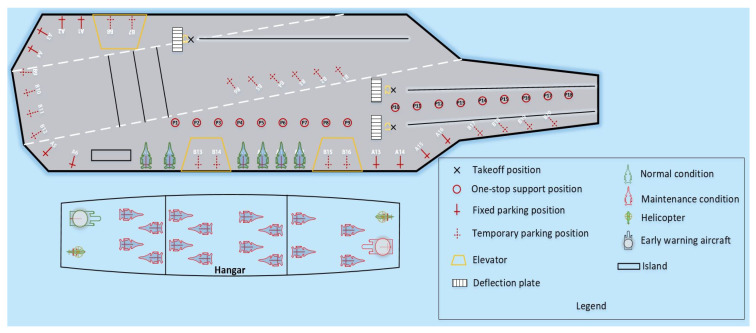
Resource model of carrier-based aircraft departure operations.

The environmental resource model (Figure 2) includes deck, hangar, shipboard aircraft, tractors, parking positions, support positions, take-off positions, deflector plates, and catapults. After integrating the current mainstream aircraft carrier deck shapes, carrier-based aircraft shapes, and support and take-off models, a 2D simulation model of the deck operations was established, including 18 one-stop support positions [16], laying a foundation for visualizing the entire scheduling process for sortie departure operations. More details can be found in Figure 2. The one-stop maintenance position enables carrier-based aircraft to complete all pre-takeoff maintenance and support procedures at one location, eliminating the need to transfer between multiple different types of maintenance positions as in the past. This significantly reduces the time required for conventional support operation and represents the current design trend for advanced aircraft carriers and the future direction for unmanned aircraft carriers.

#### 2.3.2. Collision Detection System

**Figure 3:**
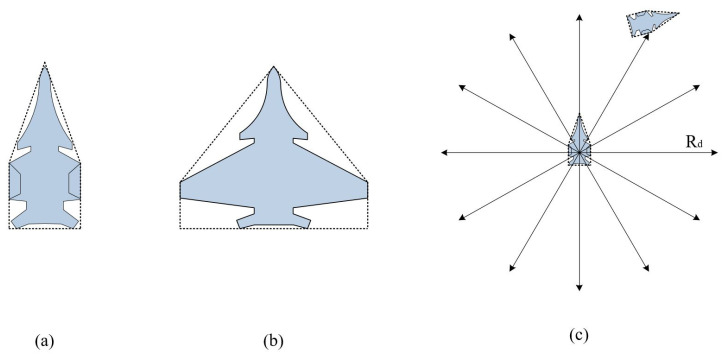
Pentagonal outer-contour shell model of the aircraft in the (**a**) folded wing state, (**b**) extended wing state, and (**c**) collision detection model of the carrier-based aircraft.

The basic collision model of carrier-based aircraft is constructed using the convex hull of the aircraft’s outer contour [17] and ray scanning [18], which is used to perceive obstacles in the deck operation environment, as shown in Figure 3. Under the convex-hull-model conditions, only the Euclidean distance between the aircraft and the target point needs to be calculated to determine whether there is a collision, significantly reducing the problem of increased computational load due to differences in the shape boundaries of different carrier-based aircraft models. Equation (1) is the condition formula for triggering the above judgment. Figure 3a,b show the pentagonal outer-contour shell model of the carrier-based aircraft in the folded and extended wing states, respectively. Figure 3c shows the collision detection model of the carrier-based aircraft. During the deck transportation process, 12 rays are formed with the geometric center point of the 2D model of the carrier-based aircraft as the center and a radius of three times the fuselage length to surround the aircraft and perceive the surrounding obstacles.

(1) $$\begin{array}{l} d_{S}=\left\{\begin{array}{l} 2l_{F}+1.5l_{C},\mathrm{Stage}\;\mathrm{of}\;\mathrm{transfer}\\ 2l_{F}+l_{W},\mathrm{Stage}\;\mathrm{of}\;\mathrm{taxiing} \end{array}\right.\\ R_{d}=\xi \cdot d_{S} \end{array},$$

where ξ denotes the deck-specific safety coefficient, $\xi \in \left[1,2\right]$.

### 2.4. Collision Avoidance Strategies for Multi-Aircraft Coordination

Given the constrained deck space, the following hierarchical strategies can address collision risks during concurrent aircraft movements:

#### 2.4.1. Basal Path Selection Protocol

Leveraging our prior inverse reinforcement learning (IRL) framework [19], we utilize a validated path repository containing the following:

- Geo-spatial way-points (x, y coordinates);
- Orientation angles at critical maneuvering nodes;
- Temporal sequencing between consecutive way-points;
- Velocity modulation patterns across path segments;
- Integrated metrics for path length and execution duration.

In this work, these baseline trajectories were extended and refined through advanced collision avoidance strategies and dynamic priority mechanisms, thereby enhancing the operational applicability of the repository in complex multi-aircraft scheduling scenarios. This evolutionary approach ensures the availability of both theoretically sound and practically executable path alternatives while maintaining continuity with our previously validated methodologies.

#### 2.4.2. Collision Avoidance Strategies for Aircraft Encounters

**Figure 4:**
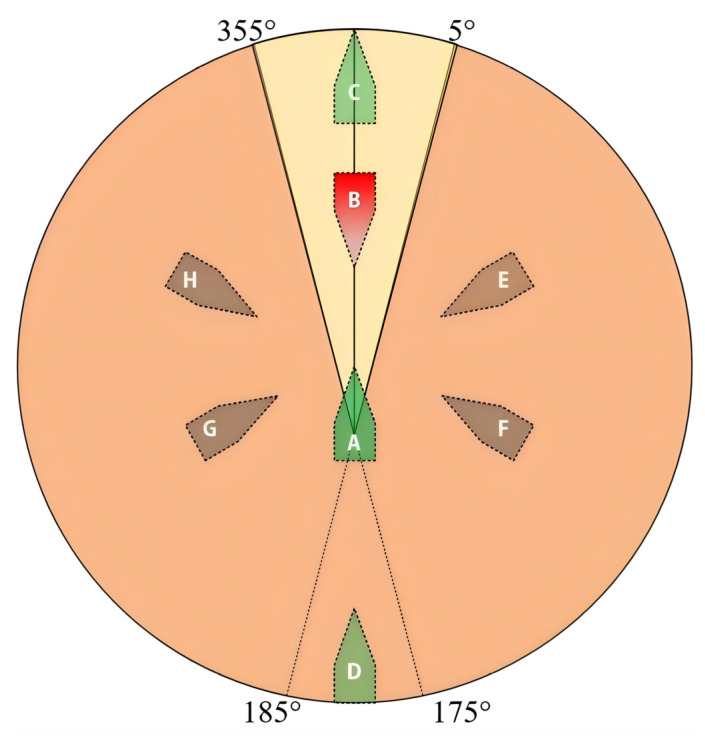
Encounter model of carrier-based aircraft deck operation.

During multi-aircraft departure operations, three encounter scenarios may occur between moving aircraft, as shown in Figure 4.

(a) Following scenario (A → C/D in Figure 4)

Priority-based yielding is implemented in constrained deck environments where overtaking is prohibited. Its strategy can be described as in Equation (2):

(2) $$\begin{array}{l} \left|Angle\_p_{i}-Angle\_p_{j}\right|\in \left[0{}^{\circ},5{}^{\circ}\right]\cup \left[355{}^{\circ},360{}^{\circ}\right],\\ d_{s}=\left\{\begin{array}{l} <\left|P_{i}P_{j}\right|,v_{i}=v_{j}\\ =\left|P_{i}P_{j}\right|,v_{i}=v_{i},v_{j}=v_{j}-\Delta v_{j}\\ >\left|P_{i}P_{j}\right|,\left(v_{i}=v_{i},v_{j}=0\right)\cup \left(v_{i}=0,v_{j}=v_{j}\right) \end{array}\right. \end{array},$$

Here, $\left|P_{i}P_{j}\right|$ represents the straight-line distance between the aircraft and the nearest point on the geometric outer contour. When $d_{s}>\left|P_{i}P_{j}\right|$, it is further determined which of the two aircraft in the encounter will adopt the waiting and evasive strategy through priority. That is, at this time, the aircraft with higher priority continues to move, while the other waits in place.

(b) Head-on scenario (A → B in Figure 4)

Conflicting aircraft execute synchronized maneuvers; this strategy can be expressed as in Equation (3):

(3) $$\begin{array}{l} \left|Angle\_p_{i}-Angle\_p_{j}\right|\in \left[175{}^{\circ},180{}^{\circ}\right]\cup \left[180{}^{\circ},185{}^{\circ}\right],\\ d_{s}=\left\{\begin{array}{l} <\left|P_{i}P_{j}\right|,\left(v_{i}=v_{j},\theta_{i}=\theta_{i}+\Delta \theta_{i},\theta_{j}=\theta_{j}+\Delta \theta_{j}\right)\\ =\left|P_{i}P_{j}\right|,\left(v_{i}=v_{i}-\Delta v_{i},v_{j}=v_{j}-\Delta v_{j},\theta_{i}=\theta_{i}+\Delta \theta_{i},\theta_{j}=\theta_{j}+\Delta \theta_{j}\right)\\ >\left|P_{i}P_{j}\right|,\left(v_{i}=v_{i}-\Delta v_{i},v_{j}=v_{j}-\Delta v_{j},\theta_{i}=\theta_{i}+\Delta \theta_{i\max},\theta_{j}=\theta_{j}+\Delta \theta_{j\max}\right) \end{array}\right. \end{array},$$

Here, $\Delta v_{i},\Delta v_{j},\Delta \theta_{i},\Delta \theta_{j}$ are the increments in the speed and steering angle of the carrier-based aircraft during movement. When the safety distance is gradually reduced, encountering carrier-based aircraft avoid collision by decelerating and increasing the steering angle on the same side.

(c) Crossing scenario (A → E/F/G/H in Figure 4)

For intersecting trajectories with maximum collision risk:

- Mandatory velocity reduction
- Differential steering governed by Equations (4) and (5):

(4) $$\begin{array}{l} \left|Angle\_P_{i}-Angle\_P_{j}\right|\in \left(5{}^{\circ},175{}^{\circ}\right),\\ d_{s}=\left\{\begin{array}{l} <\left|P_{i}P_{j}\right|,\left(v_{i}=v_{i},v_{j}=v_{j}-\Delta v_{j},\theta_{i}=\theta_{i},\theta_{j}=\theta_{j}-\Delta \theta_{j}\right)\\ =\left|P_{i}P_{j}\right|,\left(v_{i}=v_{i},v_{j}=v_{j}-\Delta v_{j},\theta_{i}=\theta_{i}-\Delta \theta_{i},\theta_{j}=\theta_{j}-\Delta \theta_{j}\right)\\ >\left|P_{i}P_{j}\right|,\left(v_{i}=v_{i},v_{j}=0,\theta_{i}=\theta_{i}-\Delta \theta_{i\max},\theta_{j}=\theta_{j}-\Delta \theta_{j\max}\right) \end{array}\right. \end{array},$$

(5) $$\begin{array}{l} \left|Angle\_P_{i}-Angle\_P_{j}\right|\in \left(185{}^{\circ},355{}^{\circ}\right),\\ d_{s}=\left\{\begin{array}{l} <\left|P_{i}P_{j}\right|,\left(v_{i}=v_{i},v_{j}=v_{j}-\Delta v_{j},\theta_{i}=\theta_{i},\theta_{j}=\theta_{j}+\Delta \theta_{j}\right)\\ =\left|P_{i}P_{j}\right|,\left(v_{i}=v_{i},v_{j}=v_{j}-\Delta v_{j},\theta_{i}=\theta_{i}+\Delta \theta_{i},\theta_{j}=\theta_{j}+\Delta \theta_{j}\right)\\ >\left|P_{i}P_{j}\right|,\left(v_{i}=v_{i},v_{j}=0,\theta_{i}=\theta_{i}+\Delta \theta_{i\max},\theta_{j}=\theta_{j}+\Delta \theta_{j\max}\right) \end{array}\right. \end{array},$$

#### 2.4.3. Obstacle Avoidance Protocol

**Figure 5:**
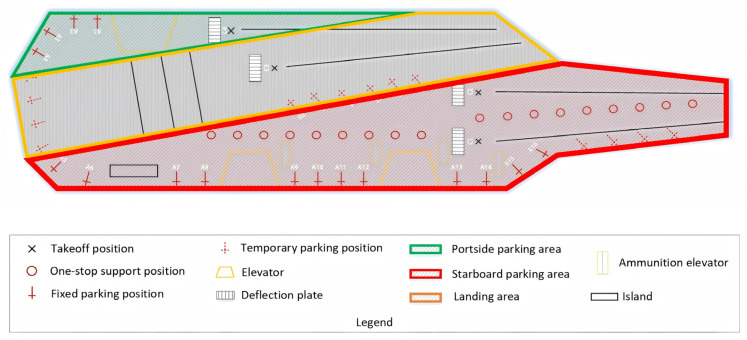
Deck obstacle and work area division diagram for an aircraft carrier.

Static/dynamic obstacles (deck edges, islands, parked aircraft) trigger:

When the obstacle potentially colliding with the carrier-based aircraft is the outer outline of the deck itself, as shown in Figure 5, the following three cases can be considered:

(1). When the aircraft is located within the port-side area of the deck (Lp), with its coordinate being $P_{i}$, its collision avoidance strategy is determined based on Equation (6).

(6) $$P_{i}\in Lp,d_{S\min}\left\{\begin{array}{l} <d_{SLi}\leq d_{S},\left(v_{i}=v_{i}-\Delta v_{i},\theta_{i}=\theta_{i}+\Delta \theta_{i}\right)\\ =d_{SLi},\left(v_{i}=0,\mathrm{Wait}\;\mathrm{for}\;\mathrm{rescheduling}\right) \end{array}\right.,$$

(2). When the aircraft is located within the starboard-side area of the deck (Rp), with its coordinate being $P_{i}$, its collision avoidance strategy is determined based on Equations (7).

(7) $$P_{i}\in Rp,d_{S\min}\left\{\begin{array}{l} <d_{SRi}\leq d_{S},\left(v_{i}=v_{i}-\Delta v_{i},\theta_{i}=\theta_{i}-\Delta \theta_{i}\right)\\ =d_{SRi},\left(v_{i}=0,\mathrm{Wait}\;\mathrm{for}\;\mathrm{rescheduling}\right) \end{array}\right.,$$

(3). When the carrier-based aircraft is in the deck landing area (Mp), it will not collide with any physical boundary on the deck. Therefore, its motion remains unchanged as expressed in Equations (8).

(8) $$P_{i}\in Mp,\left(v_{i}=v_{i},\theta_{i}=\theta_{i}\right),$$

In the case of obstacles that can potentially collide with other aircraft, tractors, or other shipboard equipment, the situation can be divided into the following:

(4). When the obstacle ($P_{Fj}$) is not a deck entity boundary and is located in the port-side area of the deck, then it satisfies the following expression:

$$P_{Fj}\in P\_CF\in Lp,\left(d_{F\min}=d_{S\min},d_{FLi}=d_{SLi},\mathrm{it}\prime s\;\mathrm{handled}\;\mathrm{according}\;\mathrm{to}\;\mathrm{Equation}\;(6)\right)$$

(5). When the obstacle ($P_{Fj}$) is not a deck entity boundary and is located in the starboard area of the deck, then it satisfies the following expression:

$$P_{Fj}\in P\_CF\in Rp,\left(d_{F\min}=d_{S\min},d_{FRi}=d_{SRi},\mathrm{it}\prime s\;\mathrm{handled}\;\mathrm{according}\;\mathrm{to}\;\mathrm{Equation}(7)\right)$$

(6). When the obstacle is not a deck entity boundary and is located within the landing area of the deck, the judgment should be made based on the relative distance between the current aircraft ($P_{i}$) and the encountered aircraft ($P_{Fj}$), as expressed in Equation (9).

(9) $$P_{Fj}\in P\_CF\in Mp,d_{FMi}=\left\{\begin{array}{l} \left|P_{i}P_{Fj}\right|>d_{S},\left(v_{i}=v_{i}-\Delta v_{i},\theta_{i}=\theta_{i}\pm \Delta \theta_{i}\right)\\ \left|P_{i}P_{Fj}\right|\leq d_{S},\left(v_{i}=v_{i}-\Delta v_{i},\theta_{i}=\theta_{i}\pm \Delta \theta_{i\max}\right)\\ \left|P_{i}P_{Fj}\right|=d_{S\min},\left(v_{i}=0,\mathrm{Wait}\;\mathrm{for}\;\mathrm{rescheduling}\right) \end{array}\right.,$$

#### 2.4.4. Dynamic Priority Assignment

Carrier-based aircraft are initially assigned departure priorities based on mission requirements. However, sole reliance on these pre-assigned priorities [20] fails to effectively coordinate multi-aircraft operations in dynamic deck environments, compromising operational efficiency and introducing safety hazards. Although collision avoidance measures (e.g., path replanning, velocity modulation, and course correction) mitigate conflicts, residual scheduling collisions persist. Ad hoc rescheduling for conflict resolution substantially degrades operational throughput and predisposes subsequent optimization processes to local optima. To address these limitations, this study develops an adaptive and real-time priority allocation mechanism that dynamically resolves scheduling conflicts during coordinated multi-aircraft operations.

- Definition of initial priority

Typically, carrier-based aircraft departure priorities are primarily determined by their type and mission profiles, as detailed in Table 1. Within the same aircraft category, ground attack missions receive higher priority than air-to-air combat or routine patrols due to their increased armament payloads and extended operational range requirements.

As specified in Table 1, the initial priority of a carrier-based aircraft scheduled for departure operations is quantified using Equation (10), where a higher summation value corresponds to elevated mission priority.

(10) $${\mathrm{Pr}}_{i}^{O}={\mathrm{Pr}}_{Ci}+{\mathrm{Pr}}_{Mi},$$

- Definition of dynamic priority

The dynamic priority of carrier-based aircraft during departure operations is computed through three critical parameters:

Total travel path length:

(11) $$\left\{\begin{array}{l} S_{i}>S_{j},\left({\mathrm{Pr}}_{Si}=0,{\mathrm{Pr}}_{Sj}=1\right)\\ S_{i}\leq S_{j},\left({\mathrm{Pr}}_{Si}=1,{\mathrm{Pr}}_{Sj}=0\right) \end{array}\right.,$$

Accumulated waiting duration:

(12) $$\left\{\begin{array}{l} T_{Wi}>T_{Wj},\left({\mathrm{Pr}}_{Wi}=1,{\mathrm{Pr}}_{Wj}=0\right)\\ T_{Wi}\leq T_{Wj},\left({\mathrm{Pr}}_{Wi}=0,{\mathrm{Pr}}_{Wj}=1\right) \end{array}\right.,$$

Remaining path ratio:

Let $\mathrm{Remaining}\;\mathrm{Path}\;\mathrm{Ratio}\left(S_{Ri}\right)=\frac{\mathrm{Length}\;\mathrm{of}\;\mathrm{aircraft}\;\mathrm{remaining}\;\mathrm{distance}}{\mathrm{Total}\;\mathrm{length}\;\mathrm{of}\;\mathrm{aircraft}\;\mathrm{path}}=\frac{S_{Ci}}{S_{i}}$, then the relationship between the remaining distance ratio and the priority can be expressed as in Equation (13):

(13) $$\left\{\begin{array}{l} S_{Ri}>S_{Rj},\left({\mathrm{Pr}}_{SRi}=0,{\mathrm{Pr}}_{SRj}=1\right)\\ S_{Ri}\leq S_{Rj},\left({\mathrm{Pr}}_{SRi}=1,{\mathrm{Pr}}_{SRj}=0\right) \end{array}\right.,$$

The composite dynamic priority, defined by Equation (14), increases with higher computed values. During conflict resolution, aircraft with elevated priority retain operational continuity, mandating collision avoidance actions from lower-priority counterparts.

(14) $${\mathrm{Pr}}_{i}={\mathrm{Pr}}_{i}^{O}+{\mathrm{Pr}}_{Si}+{\mathrm{Pr}}_{Wi}+{\mathrm{Pr}}_{SRi},$$

#### 2.4.5. Rescheduling Protocol

Should persistent collisions occur after implementing avoidance strategies, the following protocol is activated:

- Priority-driven re-queuing: Aircrafts with lower priority are re-queued according to the established hierarchy, resetting their scheduling sequence to the terminal position.
- Conflict flagging: Higher-priority aircrafts maintain operational continuity while receiving conflict markers (denoted by $C_{i}$) for trajectory re-calibration.

### 2.5. Two-Stage Scheduling Optimization Framework for Aircraft Departure Operations

Based on defined operational characteristics, the scheduling optimization model incorporates the following assumptions:

(1) Non-interruptible process: Departure operations proceed without suspension.
(2) Predetermined positional states: Deployment coordinates for aircraft and support/parking/launch positions are predefined.
(3) Persistent operational readiness: All equipment maintain baseline functionality throughout operations.
(4) Exclusive tractor assignment: Each tractor tows a single aircraft with independent workflows.
(5) Aircraft-specific timing: Warm-up, wake separation, and pre-launch durations are type-dependent constants.

To reflect the operational integrity and deck space characteristics of the aircraft, a two-stage scheduling optimization model is proposed. First, the rationality of the station selection and departure order decision in departure operation scheduling should be ensured. Second, considering the dynamic changes in the deck space and the characteristics of multi-aircraft cooperative operation, the rationality of the carrier-based aircraft scheduling path in sortie and departure operations should be ensured. For details, see Equations (15) and (16)–(27) as the constraint conditions.

(15) $$\min\left(G\right)=\min\left(\lambda_{1}\cdot T_{\max}+\lambda_{2}\cdot Conflict+\lambda_{3}\cdot Var\left(L\right)+\lambda_{4}\cdot Control\right),$$

S.→t.

(16) $$\begin{array}{l} \min\left(T_{\max}\right)\\ =\min\left(T\_tractor_{\max}+T\_standby_{\max}+T\_taxiing_{\max}+T\_preflight_{\max}+T\_catapult_{\max}\right) \end{array}$$

(17) $$\lambda_{2}\cdot conflict=\lambda_{2}^{Cataplut}\cdot \sum_{k=1}^{M}\sum_{i=1}^{K_{i}-1}\max\left(0,\Delta T^{Cataplut}-\left(t_{k,i}+t_{k,i+1}\right)\right)+\lambda_{2}^{Collision}\cdot \sum_{i=1}^{N}C_{i}$$

(18) $$C_{i}=\left\{\begin{array}{l} 0,\mathrm{no}\;\mathrm{collision}\;\mathrm{in}\;\mathrm{the}\;\mathrm{path}\;\mathrm{of}\;\mathrm{aircraft}\;i\\ 1,\mathrm{collision}\;\mathrm{occurred} \end{array}\right.$$

(19) $$Var\left(L\right)=\frac{1}{N}{\sum_{i=1}^{N}\left(L_{i}-L_{average}\right)}^{2}$$

(20) $$L_{i}=\left\{\begin{array}{l} L_{i},\mathrm{In}\;\mathrm{the}\;\mathrm{coordinated}\;\mathrm{collision}\;\mathrm{avoidance}\;\mathrm{strategy},\;\mathrm{the}\;\mathrm{aircraft}\;i\;\mathrm{does}\;\mathrm{not}\;\mathrm{need}\;\mathrm{to}\;\mathrm{wait}\\ L_{i}+\Delta l_{i},\mathrm{In}\;\mathrm{the}\;\mathrm{coordinated}\;\mathrm{collision}\;\mathrm{avoidance}\;\mathrm{strategy},\;\mathrm{the}\;\mathrm{aircraft}\;i\;\mathrm{does}\;\mathrm{not}\;\mathrm{need}\;\mathrm{to}\;\mathrm{wait} \end{array}\right.$$

(21) $$L_{average}=\frac{1}{N}\sum_{i=1}^{N}L_{i}$$

(22) $$\Delta l_{i}=T_{Wi}\cdot v_{i}$$

(23) $$Control=\frac{1}{T_{i}}\int_{0}^{T_{i}}\left(\lambda_{speed}\cdot \left|a_{speed}\left(t\right)\right|+\lambda_{steer}\cdot \left|\psi_{steer}\left(t\right)\right|\right)dt$$

(24) $${\mathrm{Pr}}_{i}>{\mathrm{Pr}}_{j}\Rightarrow T\_off_{k,i}<T\_off_{k,j}$$

(25) $$\sum_{i=1}^{N_{p}}x_{i,Pp}\leq 1,\mathrm{and}\;\forall Pp\in \left\{\mathrm{The}\;\mathrm{set}\;\mathrm{of}\;\mathrm{parking}\;\mathrm{position}\;\mathrm{of}\;\mathrm{aircraft}\right\},\exists x_{i,Pp}\in \left\{0,1\right\}$$

(26) $$\forall i\neq j,\mathrm{When}\;\mathrm{aircraft}\;i\;\mathrm{is}\;\mathrm{behind}\;\mathrm{aircraft}\;j,\;\mathrm{then}\left|t_{i}-t_{j}\right|\geq \Delta T_{wake}$$

(27) $$\min\left(T_{\max}\right)\leq T_{Base}$$

Here, G represents the total dispatch completion time for the entire departure operation, $\lambda_{1}\sim \lambda_{4}$ represent the weight coefficients of each sub-item in the target optimization function, which is used to reflect the importance of the optimization target; Equation (16) is the form of the optimization function for the scheduling duration of carrier-based aircraft departure operations, where $T\_tractor_{\max}$ is the maximum towing time from the support position to the parking position of the aircraft; $T\_standby_{\max}$ is the maximum waiting time at the parking position; $T\_taxiing_{\max}$ is the maximum taxiing time from the parking position to the take-off waiting position; $T\_preflight_{\max}$ is the maximum waiting time at the take-off waiting position for the aircraft to complete several pre-takeoff procedures such as waiting for the deflector to cool and reset, entering the take-off position, connecting the catapult device, and pre-flight inspection.

Since these procedures have strict precedence and subsequent relationships, and there are no other aircrafts interfering after entering the take-off waiting position, the completion time of these procedures is collectively represented; $T\_catapult_{\max}$ is the maximum take-off time of the aircraft on the catapult device, comprising five parts; Equation (17) is the joint penalty term in the optimization process of departure operation scheduling, that is, when the time ($t_{k,i+1}$) interval between the arrival of the aircraft (i+1) at the take-off waiting position (k) and the catapult time ($t_{k,i}$) of the preceding aircraft is too small, less than the standard interval time ($\Delta T^{catapult}$), a penalty will be triggered; a penalty is also triggered if a collision occurs during its scheduling path. Equation (18) is the constraint condition of the collision penalty indicator function; Equation (19) is the sum of variances of the aircraft paths in the current scheduling plan, used to reflect the balance of the scheduling paths; Equations (20)–(22) are the constraint conditions for the sum of variances of the aircraft scheduling paths; Equation (23) is the aircraft path control smoothing function; Equation (24) is the consistency constraint for the aircraft departure operation scheduling, that is, if aircraft i is at the takeoff position k and its dynamic priority is higher than aircraft j, then its catapult departure time will be earlier; $T\_off_{k,i}<T\_off_{k,j}$, $T\_off_{k}$ represents the actual departure time of the aircraft on the catapult runway k; Equation (25) is the uniqueness constraint for deck parking position allocation, that is, at most one aircraft can be parked at each parking position; Equation (26) is the wake interval constraint during the aircraft scheduling process; Equation (27) is the overall time constraint of the aircraft departure operation scheduling.

The $T\_catapult_{\max}$ term in Equation (16) can be disregarded in actual optimization as the catapult take-off process is typically completed within a few seconds and is related to its mechanical device, which is outside the scope of scheduling optimization. Therefore, Equation (16) can be updated to Equation (28):

(28) $$\min\left(T_{\max}\right)=\min\left(T\_tractor_{\max}+T\_standby_{\max}+T\_taxiing_{\max}+T\_preflight_{\max}\right)$$

## 3. Design of a Two-Stage Scheduling Model Solving Algorithm for Carrier-Based Aircraft Departure Operations

Existing research on carrier-based operations frequently neglects critical deck spatial characteristics or isolates specific problems such as scheduling resource allocation [21] and path planning [22]. However, practical operational scenarios necessitate integrated scheduling–path coordination. Although heuristic-based optimization methods demonstrate efficacy for small-scale scheduling, their performance degrades significantly under randomly scaled scenarios with dynamic parameters, often yielding suboptimal efficiency and solution quality.

Recent advances in deep architectures have substantially enhanced deep reinforcement learning (DRL) performance across industrial domains [23,24,25,26,27]. While prior investigations on deck scheduling paths [19] acquired feasible trajectories for carrier-based aircraft operations, most paths originated from imitation learning in constrained scenarios—limited to single-aircraft scheduling, static obstacle avoidance, or isolated encounter resolution. Consequently, these paths fail to capture spatial dynamics and multi-aircraft coordination imperatives inherent to departure operations. Nevertheless, this study repurposes prior path data as an alternative library and initial training corpus for DRL. These trajectories populate an experience replay pool, enabling DRL-based refinement of multi-aircraft collaborative scheduling paths. Furthermore, we retain the particle swarm optimization (PSO) algorithm [28,29,30]—widely adopted for sequence decision-making—to resolve the following:

(1) Aircraft parking position assignment;
(2) Take-off station selection;
(3) Take-off sequence determination;

Section 3.1 details DRL implementation with problem-specific adaptations, while Section 3.2 elaborates the enhanced PSO framework.

### 3.1. Design of AAE–SAC Algorithm

#### 3.1.1. Design of State Space, Action Space, and Reward Function

The trajectory optimization problem in carrier-based aircraft departure operations can be considered a Markov decision process (MDP). In this study, since a 2D continuous visualization simulation was adopted to study scheduling optimization problems, the state transition of the MDP was implicitly realized through dynamic changes in the deck operation environment in the simulation system, rather than through a conventional explicit structure of the state transition matrix. Therefore, the core of this problem was to reasonably design and describe its state space (S), action space (A), and reward function (R).

Under the premise of carrier-based aircraft departure operations in coordination, the state space must consider the state of the aircraft itself, as well as the states of at least two aircrafts around it, and the environmental information of the deck space. Therefore, the state space can be expressed as in Equation (29):

(29) $$S=\left[\underset{5\mathrm{Dimension}}{\underset{︸}{\mathrm{State}\;\mathrm{of}\;\mathrm{itself}}},\underset{10\mathrm{Dimension}}{\underset{︸}{\mathrm{State}\;\mathrm{of}\;\mathrm{other}\;\mathrm{aircraft}}},\underset{6\mathrm{Dimension}}{\underset{︸}{\mathrm{Environmental}\;\mathrm{information}}}\right],$$

Here, the self-state includes the current position coordinates $\left(x_{i},y_{i}\right)$ of aircraft, the speed $v_{i}$, the heading angle $\theta_{i}$, and the dynamic priority ${\mathrm{Pr}}_{i}$; the environmental information includes the coordinates $\left(x_{obs},y_{obs}\right)$ of the nearest obstacle to the aircraft, its target position coordinates $\left(x_{t\arg et},y_{t\arg et}\right)$, and the direction-angle deviation $\left(\Delta \varphi_{x},\Delta \varphi_{y}\right)=\left(\sin\left(\theta_{i}-\theta_{t\arg et}\right),\cos\left(\theta_{i}-\theta_{t\arg et}\right)\right)$ between the current position and the target position of the aircraft.

In the two-stage scheduling optimization problem of departure operations on an aircraft carrier, the first stage generates and optimizes the station position and take-off sequence problem in its scheduling scheme, while the path planning problem in the second stage runs through the entire departure operation from beginning to end. Therefore, the action at any moment in the MDP can be represented by the acceleration and angular acceleration of the carrier-based aircraft, as expressed in Equation (30):

(30) A=aspeedψsteer,and aspeed∈−0.5,0.5ms2ψsteer∈−30,30°s,

Although preliminary work has obtained the basic path library and a clear collision avoidance strategy, achieving better and more realistic new paths in the simulation optimization process that are closer to the actual situation of departure operations is anticipated. Therefore, the design of the reward function should not only consider the completion time reward of the path but also consider the quality of the path, priority, and safety in collaborative operation, etc., which can be expressed as in Equation (31):

(31) $$R=\sum_{i=1}^{N}\left[\alpha \cdot r_{time}\left(T_{i}\right)+\beta \cdot r_{path}^{\left(i\right)}+\delta \cdot {\mathrm{Pr}}_{k}\cdot r_{priority}\left(T_{i}\right)+\varepsilon \cdot r_{safety}^{\left(i\right)}\right],$$

(32) $$\alpha +\beta +\delta +\varepsilon =1,\left(\alpha ,\beta ,\delta ,\varepsilon \right)\in \left(0,1\right),$$

(33) $$r_{time}\left(T_{i}\right)=\left\{\begin{array}{l} e^{-{\left(T_{t}/T_{base\_alt}\right)}^{2}},T\leq T_{base\_alt}\\ -\log\left(T-T_{base\_alt}+1\right),T>T_{base\_alt} \end{array}\right.,$$

(34) $$r_{path}^{\left(i\right)}=\gamma \cdot sim\left(P_{i},P_{base\_alt}\right)+\eta \cdot \sum_{k=1}^{K}I_{new\_path},$$

(35) $$I_{new\_path}=\left\{\begin{array}{l} 1,\mathrm{When}\;\mathrm{the}\;k-\mathrm{th}\;\mathrm{sub}-\mathrm{path}\;\mathrm{becomes}\;\mathrm{the}\;\mathrm{new}\;\mathrm{path}\\ 0,Others \end{array}\right.,$$

(36) $$sim\left(P_{i},P_{base\_alt}\right)=\frac{1}{1+\min_{\pi }\sum_{\left(i,j\right)\in \pi }{\left\|\tau_{i}-\tau_{j}^{base\_alt}\right\|}^{2}},$$

(37) $$\tau_{i}=\left[\frac{\sum \Delta x}{\Delta T},\frac{\sum \Delta y}{\Delta T},\max\left(a_{t}\right),std\left(\theta_{t}\right)\right],$$

(38) $$r_{priority}\left(T_{i}\right)=\left\{\begin{array}{l} 2\left(1-\frac{T_{i}}{T_{base\_alt}}\right),T_{i}<0.9T_{base\_alt}\\ {\left(1-\frac{T_{i}}{T_{base\_alt}}\right)}^{2},Others \end{array}\right.,$$

(39) $${\mathrm{Pr}}_{k}=\left\{\begin{array}{l} 1.5,{\mathrm{Pr}}_{i}\geq 6\\ 1,{\mathrm{Pr}}_{i}\geq 4\\ 0.5,Others \end{array}\right.,$$

(40) $$r_{safety}^{\left(i\right)}=-\left[100\cdot \sum_{i\in \pi }C_{i}+20\cdot \sum_{i=1}^{N}I_{wake}^{\left(i\right)}+5\cdot \frac{d_{safe\_alt}}{d_{\min}^{\left(i\right)}}\right],$$

(41) $$I_{wake}^{\left(i\right)}=\left\{\begin{array}{l} 1,\Delta T_{\left(i,j\right)}\leq \Delta T_{wake}\\ 0,Others \end{array}\right.,$$

Equation (32) represents the weight factors for each sub-item reward. Equations (33)–(41) are the sub-item constraints of the joint reward function of Equation (31), where Equation (33) is the reward function for the completion time of the scheduling path, adopting a nonlinear formula to process it in stages, which is beneficial to improving the training efficiency and enhancing the marginal effect; Equation (34) is the reward function for exploring new paths, based on the existing paths; this paper introduces a dynamic time warping mechanism [31,32] to divide the current path into multiple sub-path segments and compare them with the basic path to improve the efficiency of exploring new paths; Equations (35)–(37) are the constraints of the reward function for exploring new paths; Equations (38) and (39) constitute the reward function for different task priorities, emphasizing the dynamic reward mechanism of prioritizing high-priority tasks while completing path planning; Equation (40) is the reward function for path safety, which includes penalties for unsafe behaviors, such as collision, wake turbulence, and safe driving distance; Equation (41) is the constraint condition of the wake turbulence penalty term.

#### 3.1.2. Dynamic Target Entropy and Attention Mechanism

**Figure 6:**
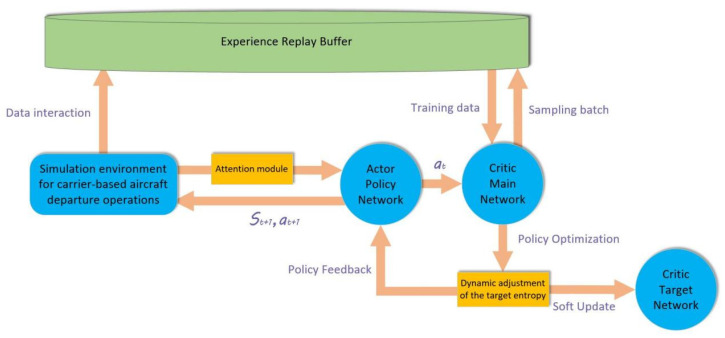
Schematic of the network structure of the AAE–SAC algorithm.

The soft actor–critic (SAC) is a reinforcement learning algorithm based on the actor and critic (A–C) framework, capable of efficiently handling optimization problems in continuous action spaces. Therefore, in this study, a deep reinforcement learning network based on the SAC algorithm was adopted to solve the multi-aircraft cooperative path planning problem in the optimization of carrier-based aircraft departure operations. However, unlike the commonly used fixed-entropy strategy, for aircraft operating in a known basic path and in a confined space, the issues of multi-aircraft cooperation and the utilization of the basic path still needs to be addressed. This makes it difficult for the fixed-entropy strategy to adapt to such a complex operational environment in terms of exploration ability and efficiency. Hence, an improvement is proposed by dynamically adjusting the target entropy [33], and its mathematical expression is expressed as in Equation (45). At the same time, an attention mechanism [34] is introduced into the algorithm, focusing on the nearest obstacle and its dynamic priority changes in the flight path of the carrier-based aircraft, thus achieving focused learning of high-dimensional key features in complex simulation environments. Compared with previous studies [35] that determined the observation focus of carrier-based aircraft departure and recovery capabilities through extensive calculations and the establishment of system evaluation indicators, the SAC algorithm improved with the attention and entropy adjustment strategy and has better adaptability and learning efficiency for the carrier-based aircraft scheduling optimization problem. This algorithm is named attentive adaptive entropy SAC (AAE–SAC) and is mathematically expressed as in Equations (42)–(44). Figure 6 shows the network structure diagram.

(42) $$\forall s\in S\in R^{21},\exists w\left(s\right)=\mathrm{Softmax}\left(W_{2}\cdot \mathrm{ReLU}\left(W_{1}s+b_{1}\right)+b_{2}\right),$$

(43) $$s_{attn}=w⊗\left[v,\theta ,p,\Delta x\right]=\left[w_{v}\cdot v,w_{\theta }\cdot \theta ,w_{p}\cdot p,w_{\Delta }\cdot \Delta x\right],$$

(44) $$w\left(s\right)\in R^{4},w=\left[w_{v},w_{\theta },w_{p},w_{\Delta }\right]\;\mathrm{and}\;\sum w_{i}=1,$$

The MDP state space of the carrier-based aircraft in the departure operation environment can be described as a 21-dimensional state space. When inputted to the SAC deep reinforcement learning network, the 21-dimensional state features can be weighted and focused on the nearest obstacle and its dynamic priority with respect to the current carrier-based aircraft through Equations (42) and (43) through the attention mechanism, thereby guiding the training direction of the actor policy network; $W_{1},W_{2},b_{1},b_{2}$ are network parameters.

(45) $$H_{t\arg et}^{\left(k\right)}\left(t\right)=H_{h\_base}^{\left(k\right)}+\beta \cdot \frac{C_{i}^{\left(k\right)}}{C_{\max}}+\alpha_{attn}\cdot \left(1-\sum w_{p}\right),$$

(46) $$H_{h\_base}^{\left(k\right)}=-\dim\left(a\right)\cdot \left(1-\frac{T_{h\_best}^{\left(k\right)}}{T_{current}^{\left(k\right)}}\right),$$

(47) $$\forall \pi \in S_{g},\exists \alpha \in R\;\mathrm{and}\;H\left(\pi_{t}\right)=E_{s\sim p^{\pi }}\left[-\log\pi \left(\left.a\right|s\right)\right],$$

(48) $$\alpha_{t+1}=\alpha_{t}\cdot \exp\left(\eta \left(H\left(\pi_{t}\right)-H_{t\arg et}^{\left(k\right)}\left(t\right)\right)\right),$$

Equation (45) is the mathematical expression for the dynamic target entropy; $\alpha_{attn}\cdot \left(1-\sum w_{p}\right)$ represents the compensation term of the policy entropy when the priority weight obtained by the current attention mechanism is insufficient; Equation (46) is the expression form of the reference entropy, the value of which is related to the dimension of the action space and the time used for scheduling; Equation (47) is the expression form of the entropy under the current policy. Through Equations (45)–(47), the update rule of the entropy coefficient in Equation (48) can be obtained, thereby achieving feedback and optimization of the policy of the actor network.

Following the above discussion, the network structure of the AAE–SAC algorithm can be summarized as mainly comprising the actor network, the critic main network, the critic target network, and the experience replay pool containing known path samples. Among them, the critic main network adopts a dual Q-network design [36], and its corresponding target network is also a dual-target network. The dual-network structure can avoid the overestimation of the Q-values and poor stability caused by noise in a single network or abnormal sample data, thereby avoiding high-risk actions during carrier-based aircraft scheduling and improving operational safety. The pseudocode specification for the AAE-SAC algorithm is formally detailed in Algorithm 1.

Specifically, the actor policy network includes an input layer, which is used to receive the state space parameters of the carrier-based aircraft sortie and departure operation simulation, and two fully connected neural networks with 64 neurons in the middle layer. The structure of the critic dual network and target network is the same, both including an output layer and two fully connected neural networks with 128 neurons. The critic target network synchronizes the parameters of the critic main network through a soft update mechanism to improve training stability; the activation function of all the above networks is the ReLU function.
**Algorithm 1.** Attentive Adaptive Entropy SAC (AAE–SAC) AlgorithmRequire: Number of aircraft: N, $\mathrm{State}\;\mathrm{space}:\;S\in R^{21}$, $\mathrm{Action}\;\mathrm{space}:\;A=\left[\begin{array}{l} a_{speed}\\ \psi_{steer} \end{array}\right]$, $\mathrm{Base}\;\mathrm{paths}:\;P_{base\_alt}$, $\mathrm{Maximum}\;\mathrm{episodes}:\;E_{\max}$ Ensure:                     $\mathrm{Optimized}\;\mathrm{policy}\;\pi^{*}$, Learned Q-functions: $Q_{\phi_{1}},Q_{\phi_{2}}$ 1: Initialize networks:
2:    $\;\mathrm{Actor}\;\pi_{\theta }$$,\;\mathrm{Critics}\;Q_{\phi_{1}},Q_{\phi_{2}}$ 3:    $\;\mathrm{Target}\;\mathrm{networks}\;{\bar{Q}}_{\phi_{1}}\leftarrow Q_{\phi_{1}},{\bar{Q}}_{\phi_{2}}\leftarrow Q_{\phi_{2}}$ 4:     Replay buffer D←∅ 5:     Temperature α←1.0 6: **for** episode e←1 to $E_{\max}$ **do** 7:         $\;\mathrm{Initialize}\;\mathrm{state}\;s_{0}$ 8:          for step t←1$\;\mathrm{to}\;T_{\max}$ **do** 9:                    Compute attention weights:                                  $w\left(s\right)\leftarrow \mathrm{Softmax}\left(W_{2}\cdot \mathrm{ReLU}\left(W_{1}s+b_{1}\right)+b_{2}\right)$ 10:                         Apply attention:                                    $s_{attn}\leftarrow \left[w_{v}\cdot v,w_{\theta }\cdot \theta ,w_{p}\cdot p,w_{\Delta }\cdot \Delta x\right]$ 11:                         Select action:                                    $a_{t}\leftarrow \pi_{\theta }\left(s_{attn}\right)+N\left(0,\sigma \right)$ 12:                        $\;\mathrm{Execute}\;a_{t}$$,\;\mathrm{observe}\;r_{t}$$\;\mathrm{and}\;s_{t+1}$ 13:                        Compute reward components:                           $r_{t}\leftarrow \sum_{i=1}^{N}\left[\alpha \cdot r_{time}\left(T_{i}\right)+\beta \cdot r_{path}^{\left(i\right)}+\delta \cdot {\mathrm{Pr}}_{k}\cdot r_{priority}\left(T_{i}\right)+\varepsilon \cdot r_{safety}^{\left(i\right)}\right]$ 14:                       $\;\mathrm{Store}\;\mathrm{transition}\;\left(s_{t},a_{t},r_{t},s_{t+1}\right)$ in D 15:                          **if** update time **then** 16:                                Sample batch from D 17:                                Compute target Q-values:                                      $\tilde{Q}\leftarrow r+\gamma \underset{j=1,2}{\min}\;{\bar{Q}}_{\phi_{j}}\left(s^{\prime },\pi_{\theta }\left(s^{\prime }\right)\right)-\alpha \log\pi_{\theta }\left(a^{\prime }\left|s^{\prime }\right.\right)$ 18:                                   Update critics:                                                   $\phi_{j}\leftarrow \phi_{j}-\eta_{Q}\nabla_{\phi_{j}}\frac{1}{\left|B\right|}\sum {\left(Q_{\phi_{j}}\left(s,a\right)-\tilde{Q}\right)}^{2}$ 19:                                   Update policy:                                   $\theta \leftarrow \theta -\eta_{\pi }\nabla_{\theta }\frac{1}{\left|B\right|}\sum \left(\alpha \log\pi_{\theta }\left(a\left|s\right.\right)-\underset{j=1,2}{\min}Q_{\phi_{j}}\left(s,\pi_{\theta }\left(s\right)\right)\right)$ 20:                                   Update temperature:                                                  $\alpha_{t+1}\leftarrow \alpha_{t}\cdot \exp\left(\eta \left(H\left(\pi_{t}\right)-H_{t\arg et}^{\left(k\right)}\left(t\right)\right)\right)$ 21:                                 **end if** 22:                     **end for** 23:       **end for** 24:  **return**
$\pi_{\theta }$$,\;Q_{\phi_{1}},Q_{\phi_{2}}$


### 3.2. Design of LTA-HPSO Algorithm

#### 3.2.1. Particle Swarm Coding and Decoding

As mentioned previously, this paper adopts the PSO algorithm to solve station selection and take-off sequencing problems in shipboard aircraft departure operations. As a swarm intelligence algorithm, PSO features simple implementation, rapid convergence, and excellent hybridization compatibility. Its explicit encoding scheme provides superior interpretability for scheduling optimization. To simultaneously capture critical information on parking positions, take-off positions, and take-off sequences, we design a hierarchical encoding method [37] as follows:

For station selection from the support position to the parking position, we suppose there are currently five parking positions available on the deck, and $x_{i}$ is a continuous-value variable reflecting the selection of the parking position by the carrier-based aircraft. If $x_{i}\in \left(0.6,\left.0.8\right]\in \left[0,1\right]\right.$, it indicates that aircraft i has chosen the fourth parking position among the five available parking positions.

For station selection from the parking position to the take-off position, we suppose there are currently three take-off positions available on the deck, and $y_{i}$ is a continuous-value variable reflecting the selection of the take-off position by the carrier-based aircraft. If $y_{i}\in \left(0.33,\left.0.66\right]\in \left[0,1\right]\right.$, it indicates that the aircraft i has chosen the second take-off position among the three available take-off positions.

The determination of the take-off sequence is not only based on the sequence of the operation times but also should fully consider the priority of the departure tasks. Therefore, the take-off sequence is related to the priority of the aircraft operation. Suppose $z_{i}$ is a variable reflecting the take-off sequence of the aircraft, and $z_{i}=\frac{1}{{\mathrm{Pr}}_{i}}\in \left(0,\left.1\right]\right.$, the lower the value, the earlier the take-off sequence.

For the departure operation of N carrier-based aircraft on the deck, the complete form of the particle swarm coding that determines the station selection and take-off sequence can be expressed as in Equation (49), with a total particle dimension of 3N.

(49) $$X=\left[\underset{\mathrm{Code}\;\mathrm{of}\;\mathrm{parking}\;\mathrm{position}}{\underset{︸}{x_{1},x_{2},\ldots ,x_{N}}},\underset{\mathrm{Code}\;\mathrm{of}\;\mathrm{take}-\mathrm{off}\;\mathrm{position}}{\underset{︸}{y_{1},y_{2},\ldots ,y_{N}}},\underset{\mathrm{Code}\;\mathrm{of}\;\mathrm{take}-\mathrm{off}\;\mathrm{sequence}}{\underset{︸}{z_{1},z_{2},\ldots ,z_{N}}}\right],$$

#### 3.2.2. Population Division

If the flight deck is considered the search space and the carrier-based aircraft as particles, then for a deck space with M parking positions, L take-off positions, and N carrier-based aircraft, its search space can be expressed as in Equation (50). In medium- and large-scale continuous scheduling of carrier-based aircraft operations, the search space will be significantly large. To reduce the computational load caused by the large search space, based on the idea of dynamic cluster division [38], the carrier-based aircraft particles in the deck space are divided into multiple sub-populations according to the task coupling degree and sub-population size control, as expressed in Equations (51)–(54); subsequently, multiple sub-populations are optimized in parallel, and the optimal solutions of each sub-population are merged to form the optimal dispatching plan for the departure of this batch of carrier-based aircraft.

(50) $$SearchSpace=M^{N}\times L^{N}\times N!,$$

(51) $$MCoupling_{ij}=\alpha \cdot \mathrm{Task}\;\mathrm{temporal}\;\mathrm{overlap}\;\mathrm{degree}\left(i,j\right)+\beta \cdot \mathrm{The}\;\mathrm{degree}\;\mathrm{of}\;\mathrm{competition}\;\mathrm{for}\;\mathrm{take}-\mathrm{off}\;\mathrm{positon}\left(i,j\right)$$

Here, α,β are the weight coefficients in the emphasis direction of the task coupling relationship, $\alpha ,\beta \in \left[0,1\right]$;

(52) $$\mathrm{Task}\;\mathrm{temporal}\;\mathrm{overlap}\;\mathrm{degree}\left(i,j\right)=\frac{\max\left(0,\min\left(Te_{i}-Te_{j}\right)-\max\left(Ts_{i}-Ts_{j}\right)\right)}{\max\left(Te_{i}-Te_{j}\right)-\min\left(Ts_{i}-Ts_{j}\right)}$$

(53) $$\mathrm{The}\;\mathrm{degree}\;\mathrm{of}\;\mathrm{competition}\;\mathrm{for}\;\mathrm{take}-\mathrm{off}\;\mathrm{position}\left(i,j\right)=\sum_{l=1}^{L}Poff_{i}\left(l\right)\cdot Poff_{j}\left(l\right)$$

(54) $$\mathrm{The}\;\mathrm{number}\;\mathrm{of}\;\mathrm{aircraft}\;\mathrm{in}\;\mathrm{the}\;\mathrm{sub}-\mathrm{population}:1\leq N\_Sub_{\max}=\left\lceil \sqrt{N}\right\rceil$$

#### 3.2.3. Fitness Function

During the scheduling process, an improved algorithm based on PSO is adopted to handle the station selection and take-off sequence decision-making problems in the operation, while the AAE–SAC algorithm solves the real-time control of the path. Therefore, it is inevitable that the scheduling plan generated by PSO cannot necessarily ensure that all carrier-based aircraft can complete the departure operation smoothly. That is, there may be conflicts when the time intervals between the arrival of certain carrier-based aircrafts at the take-off position are too small. There may also be situations where the operation is completed but does not necessarily conform to the priority order of the carrier-based aircraft. Therefore, in the design of the fitness function, it is not only necessary to consider whether the final completion time of the optimization plan is optimal but also whether the handling of high-priority tasks in the plan is reasonable and whether the take-off sequence meets the safety intervals of the take-off time. The specific form is expressed as in Equations (55)–(57).

(55) $$f=\max\left({\mathrm{Pr}}_{i}\cdot T_{i}\right)+\lambda \cdot \underset{\mathrm{Time}\;\mathrm{conflict}\;\mathrm{penalty}\;\mathrm{term}}{\underset{︸}{P_{launch}}},$$

(56) $$\lambda^{\left(t+1\right)}=\lambda^{\left(t\right)}\cdot \left(1+\sum_{l=1}^{L}Coff_{l}\right),$$

(57) $$F=\frac{1}{f},$$

Equation (55) is the expression for the fitness function variable f that reflects the relationship between priority and time conflict functions; Equation (56) is the adaptive adjustment function for the penalty time coefficient in Equation (55), that is, the greater the number of take-off interval conflicts in the current iteration process, the greater the penalty time coefficient in the next round of iteration; and Equation (57) is the fitness function expression.

#### 3.2.4. Update Mechanism

In the proposed particle update mechanism, adaptive adjustments to the learning factors in the particle velocity update are introduced (Equations (58)–(61)), replacing conventional static methods. Laplace-distributed perturbation is applied to these learning factors $\left(c_{1},c_{2}\right)$ [39], where the long-tail characteristic facilitates exploration beyond current optimal regions, thereby enhancing the algorithm’s ability to escape local optima. Concurrently, a tabu list mechanism [40] is incorporated to systematically exclude non-improving or revisited solutions during iterations (Equations (62)–(63)), significantly boosting computational efficiency while ensuring solution quality.

(58) $$v_{i}^{\left(t+1\right)}=\omega v_{i}^{\left(t\right)}+\underset{\mathrm{Individual}\;\mathrm{perturbation}}{\underset{︸}{\left(c_{1}\cdot Laplace\left(0,b\right)\right)}}\cdot \left(p_{best}-x_{i}^{\left(t\right)}\right)+\underset{\mathrm{Social}\;\mathrm{perturbation}}{\underset{︸}{\left(c_{2}\cdot Laplace\left(0,b\right)\right)}}\cdot \left(g_{best}-x_{i}^{\left(t\right)}\right)$$

(59) $$x_{i}^{\left(t+1\right)}=x_{i}^{\left(t\right)}+v_{i}^{\left(t+1\right)},$$

(60) $$\omega =\omega_{\max}-\frac{\left(\omega_{\max}-\omega_{\min}\right)\cdot t}{t_{\max}},$$

(61) $$b=\frac{t}{t_{\max}},$$

Here, $Laplace\left(0,b\right)$ is the Laplace distribution with location parameter 0 and scale parameter b; t is the current iteration number, and $t_{\max}$ is the total number of iterations.

(62) $$f\left(x_{new}\right)>\mu_{f}+\alpha \cdot \sigma_{f},$$

(63) $$T_{new}=T_{base}+\beta \cdot \left(\frac{f\left(x\right)-f_{best}}{f_{best}}\right),$$

Equation (62) represents the degree of deterioration of the solution generated in the current iteration. If the fitness value $f\left(x_{new}\right)$ of the current solution is greater than the sum of the average fitness value $\mu_{f}$ and the weighted fitness standard deviation $\alpha \cdot \sigma_{f}$ of the current population, where $\alpha \in \left[1,3\right]$ is the coefficient of solution deterioration significance, it indicates that the current solution has significantly deteriorated and should be placed in the tabu list. Equation (63) is the expression for the dynamic validity period of the tabu list, that is, the worse the current solution $f\left(x\right)$ compared with the historical optimal solution $f_{best}$, the longer the validity period of its tabu list. $T_{base}$ is the initial validity period of the tabu list, and $\beta \in \left[1,3\right]$ is the sensitivity coefficient of the dynamic validity period.

**Figure 7:**
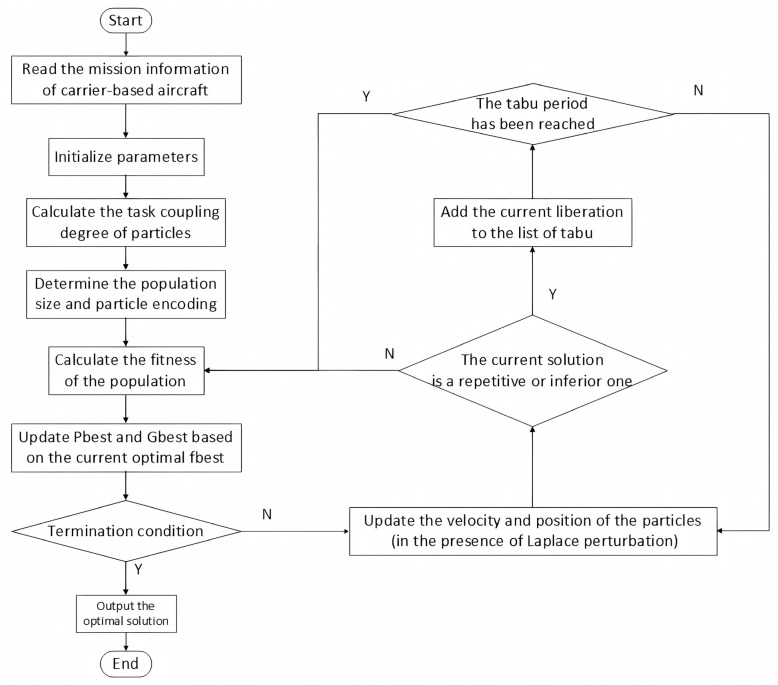
Flowchart of the LTA-HPSO algorithm.

In summary, a detailed discussion on the encoding and decoding, population division, fitness function, and particle update mechanism related to the PSO algorithm was made. Thus, the optimization algorithm LTA-HPSO was established for solving the problem of choosing the station position and determining the take-off sequence of the carrier-based aircraft. Figure 7 shows the algorithm flowchart. The pseudocode specification for the LTA-HPSO algorithm is formally detailed in Algorithm 2.
**Algorithm** **2.** Laplacian–Tabu Augmented Hierarchical PSO (LTA-HPSO) AlgorithmRequire: Number of aircraft: N              Number of parking positions: M               Number of departure positions: L               $\mathrm{Maximum}\;\mathrm{iterations}:\;t_{\max}$               $\mathrm{Initial}\;\mathrm{population}:\;X=\left[x_{1},x_{2},\ldots ,x_{N},y_{1},y_{2},\ldots ,y_{N},z_{1},z_{2},\ldots ,z_{N}\right]$               $\mathrm{Priority}\;\mathrm{of}\;\mathrm{each}\;\mathrm{aircraft}:\;{\mathrm{Pr}}_{i}$ for i=1,2,…,N Ensure: Optimal parking position selection and departure order: $X_{best}$               Minimum fitness value: $F_{best}$ 1: Initialize population X$\;\mathrm{and}\;\mathrm{velocities}\;v_{i}$ for all particles 2: Initialize $X_{best}\leftarrow X$ 3: $\mathrm{Initialize}\;F_{best}\leftarrow \infty$ 4: **for**
t←1$\;\mathrm{to}\;t_{\max}$ **do** 5:       **for** each particle i in the population **do** 6:              $\mathrm{Update}\;\mathrm{velocity}\;v_{i}$ using Laplacian perturbation: $v_{i}^{\left(t+1\right)}\leftarrow \omega v_{i}^{\left(t\right)}+\left(c_{1}\cdot Laplace\left(0,b\right)\right)\cdot \left(p_{best}-x_{i}^{\left(t\right)}\right)+\left(c_{2}\cdot Laplace\left(0,b\right)\right)\cdot \left(g_{best}-x_{i}^{\left(t\right)}\right)$ 7:              $\mathrm{Update}\;\mathrm{position}\;x_{i}$:                                                 $x_{i}^{\left(t+1\right)}\leftarrow x_{i}^{\left(t\right)}+v_{i}^{\left(t+1\right)}$ 8:               $\mathrm{Evaluate}\;\mathrm{fitness}\;F\left(x_{i}^{\left(t+1\right)}\right)$:                                             $F\left(x_{i}^{\left(t+1\right)}\right)\leftarrow \frac{1}{\max\left({\mathrm{Pr}}_{i}\cdot T_{i}\right)+\lambda \cdot P_{launch}}$ 9:                 **if**
$F\left(x_{i}^{\left(t+1\right)}\right)<F_{best}$ **then** 10:                 $X_{best}\leftarrow x_{i}^{\left(t+1\right)}$ 11:                 $F_{best}\leftarrow F\left(x_{i}^{\left(t+1\right)}\right)$ 12:              **end if** 13:          **end for** 14:          Update penalty coefficient λ:                                                   $\lambda^{\left(t+1\right)}\leftarrow \lambda^{\left(t\right)}\cdot \left(1+\sum_{l=1}^{L}Coff_{l}\right)$ 15:            Update inertia weight ω:                                                  $\omega \leftarrow \omega_{\max}-\frac{\left(\omega_{\max}-\omega_{\min}\right)\cdot t}{t_{\max}}$ 16:            Update Laplacian scale parameter b:                                                  $b\leftarrow \frac{t}{t_{\max}}$ 17:            Update tabu table: 18:            **if**
$f\left(x_{new}\right)>\mu_{f}+\alpha \cdot \sigma_{f}$ **then** 19:                     $\mathrm{Add}\;x_{new}$ to tabu table 20:           **end if**
21: **end for**
22: **return**
$X_{best},F_{best}$

### 3.3. Summary of Solution Methods

**Figure 8:**
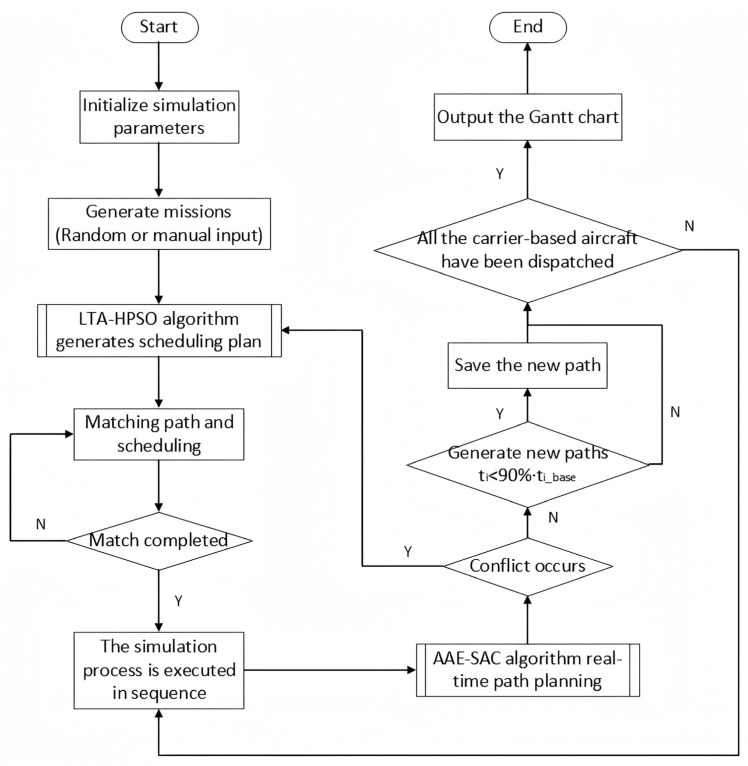
Simulation flowchart of a two-stage scheduling optimization algorithm for aircraft departure operations.

The previous two subsections expounded on the real-time planning of the scheduling path for departure operations on an aircraft carrier through deep reinforcement learning methods, establishing the AAE–SAC algorithm for solving the path planning problem. We also explained the optimization process of the decision-making schemes for the parking positions, take-off positions, and take-off sequence of the aircraft during the departure operation through the PSO method, establishing the LTA-HPSO algorithm for solving the station matching and sequence decision-making problem. Figure 8 shows a flowchart of the two-stage scheduling optimization algorithm for solving departure operations of the carrier-based aircraft. After the simulation program is started and initialization is completed, the user can choose to have the system generate combat tasks or manually define these tasks. The process of the LTA-HPSO sub-program is shown in Figure 7. The simulation system generates a scheduling plan based on the LTA-HPSO algorithm to match the corresponding station positions and then executes the simulation process in sequence. During this period, the AAE–SAC algorithm is used as a sub-program to perform real-time control of the scheduling path; its process is shown in Figure 6. If the final completion time of the real-time path in the simulation is less than or equal to 90% of the time taken by the original matching path (from [19]), a new path is considered to have been generated and is then saved. Otherwise, when the simulation reaches the termination condition, the Gantt chart of its current optimal operation process is outputted, and the simulation is terminated.

## 4. Simulation Experiment and Result Analysis

Taking a typical aircraft carrier deck as an example, this study performed simulation optimization experiments on carrier-based aircraft departure operations. The flight deck was assumed to have 18 one-stop support positions, 16 fixed parking positions (numbered A01–A16), 20 temporary parking positions (numbered B01–B20), three elevators, three catapult take-off positions, and runways. Table 2 and Table 3 present the algorithm parameters and operation-related parameters, respectively. The simulation environment was a carrier-based aircraft departure operation simulation platform developed using C + + in a Windows 10 operating system running on a PC with 8 GB of memory and a main frequency of 2.30 GHz.

### 4.1. Case 1

**Figure 9:**
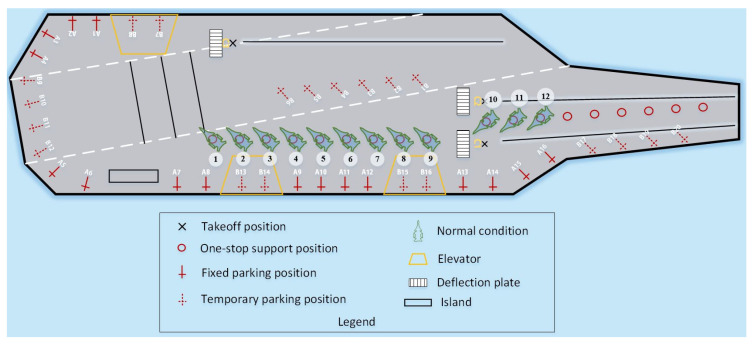
Initial station map of Case 1.

Case 1 involved 12 stealth fighter jets on the flight deck at support positions and fully supported. Some combat missions may require all existing carrier-based aircraft to be dispatched. Figure 9 shows the initial deck status and position information. Under the conditions of this case, the proposed algorithm was compared with two benchmark algorithms, namely, the conventional PSO algorithm and the deep reinforcement learning method. Each algorithm independently ran 10 simulations of the scheduling process. Table 4 presents the solution results obtained by the three algorithms for Case 1. The LTA-HPSO + AAE–SAC algorithm exhibited the best optimal scheduling time of 609 s. Table 5 presents the corresponding optimal scheduling plan.

**Figure 10:**
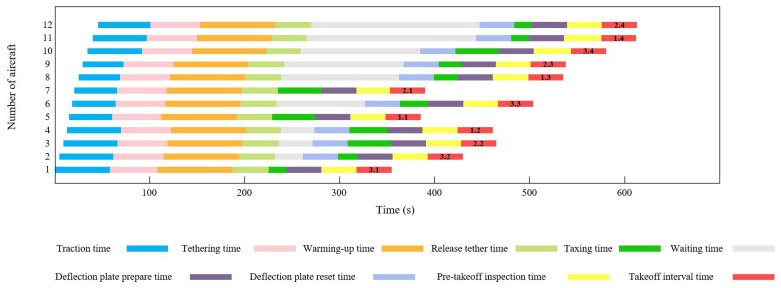
Gantt chart of 12 carrier-based aircraft departure operation.

**Figure 11:**
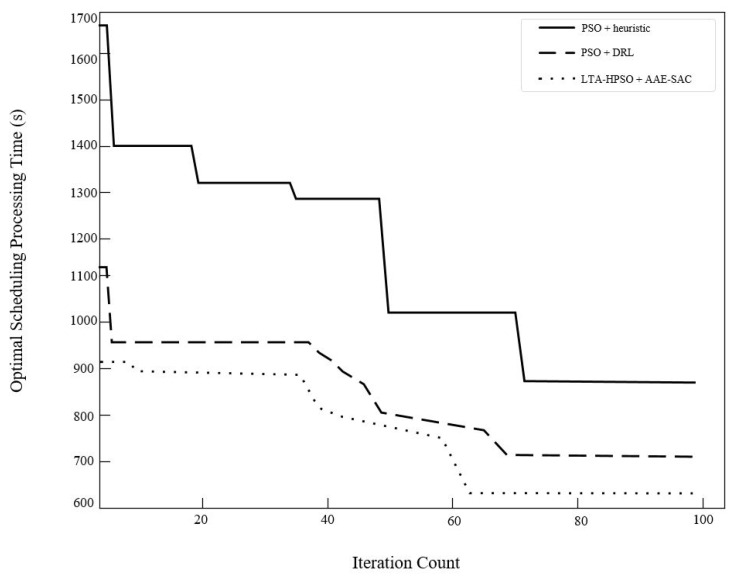
Iterative curves of the solutions obtained by three algorithms in Case 1.

The Gantt chart of the departure operation of 12 shipboard aircraft is shown in Figure 10. Below the chart is the description of the operation content represented by different color blocks. The departure operation color blocks at the end of each Gantt chart contain the take-off sequence of the carrier-based aircraft in decimal form. For example, the take-off sequence of aircraft No. 1 is 3.1, where the integer part 3 indicates that its take-off runway is located at the catapult No. C3, and the decimal part 1 indicates that it takes off in the first sequence on the C3 runway. The take-off sequence of other aircraft can be inferred in the same way. Figure 11 verifies the optimization effect of the algorithm proposed in this paper on the departure operation scheduling of carrier-based aircraft from the perspective of convergence.

### 4.2. Case 2

**Figure 12:**
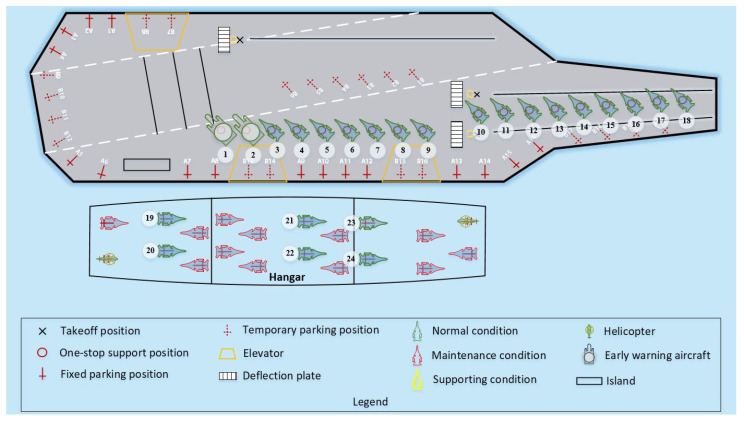
Initial station map of Case 2.

**Figure 13:**
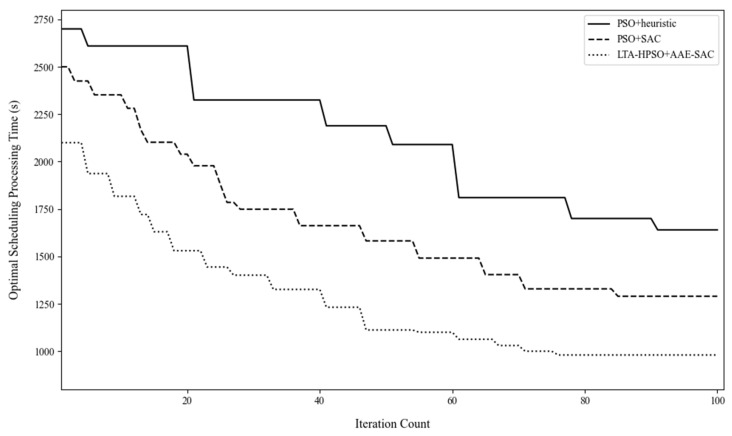
Iterative curves of the solutions obtained by three algorithms in Case 2.

**Figure 14:**
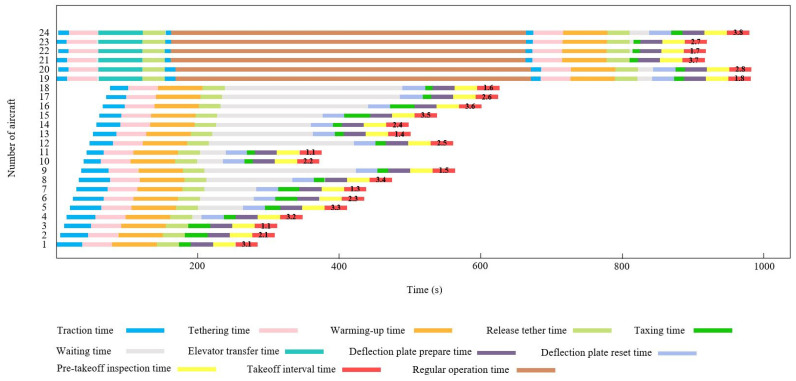
Gantt chart of 24 carrier-based aircraft departure operations.

In Case 2, the sortie scale of the carrier-based aircraft was increased to 24 to explore the ability of the LTA-HPSO + AAE–SAC algorithm in solving large-scale, multi-constraint scheduling optimization problems. Accordingly, the test design involved 18 aircraft on deck in the one-stop support position and supported, including two early warning aircraft (located at maintenance positions P1 and P2, from left to right) and two heavy carrier-based aircraft (located at maintenance positions P10 and P11). There were sufficient spare aircraft in the hangar. Figure 12 shows the initial state of the deck and position information. Under the conditions of this case, each of the three algorithms independently ran 10 simulations of the scheduling process. Table 6 presents the solution results for Case 2. Figure 13 shows the iterative curves of the solutions obtained by the three algorithms for Case 2. The LTA-HPSO + AAE–SAC algorithm required 984 s for optimal scheduling; Table 7 presents the corresponding optimal scheduling plan. Figure 14 shows the Gantt chart of the departure operation of 24 aircraft.

**Figure 15:**
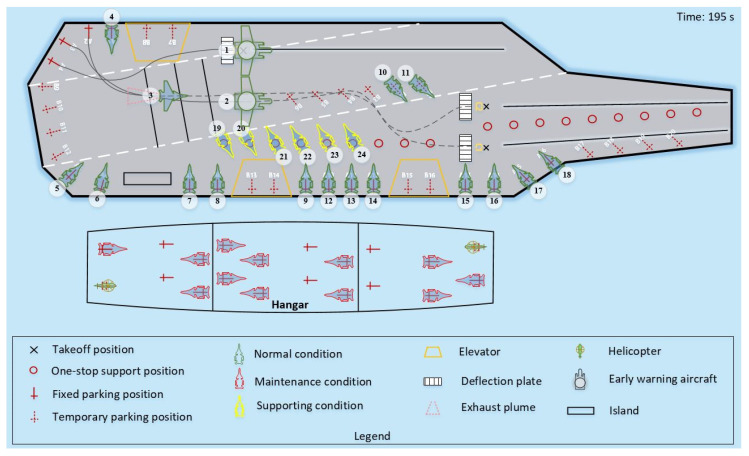
Snapshot of the departure operation simulation test with early-warning aircraft.

**Figure 16:**
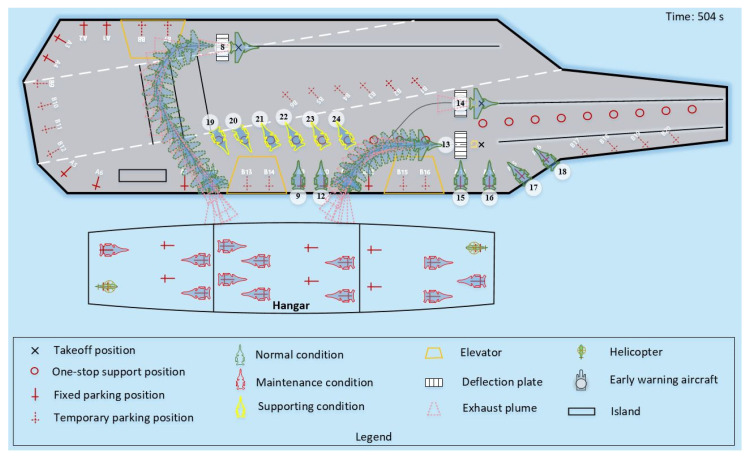
Snapshot of the simulation test for the departure operation of an aircraft with strong constraints on deck space for positions.

Case 2, in addition to having a different scale of scheduling from Case 1, also includes different types of shipboard aircraft, which makes the scheduling optimization not only need to consider the change in quantity but also deal with multiple scheduling demands such as different priority aircraft and different operation tasks. Therefore, we use Figure 15 and Figure 16 to demonstrate the LTA-HPSO + AAE-SAC algorithm’s ability to collaboratively optimize multi-type aircraft and differentiated operations in large-scale scheduling. Figure 15 shows the instantaneous image at 195 s of the simulation experiment, from which it can be seen that the algorithm prioritizes the handling of early warning aircraft with higher priority, and its scheduling path is safe and reasonable. Figure 16 reflects the collaborative path planning process of aircraft taking off and leaving the field when the station positions are strongly constrained by deck space. At 504 s of the simulation experiment, the four aircrafts (numbered: 9, 12, 13, 14) located at parking positions A9-A12 have to detour their scheduling paths due to the occupation of their forward support stations (P3-P6), and there are multiple constraints such as operation space and wake interference. Under such circumstances, the scheduling path trajectory and the aircraft’s outer contour and wake envelope line planned by the algorithm from the side reflect the algorithm’s outstanding optimization ability for the departure scheduling problem under changes in priority and operation space, and the decision path is safe and reasonable.

### 4.3. Analysis of Algorithm Performance

#### 4.3.1. Algorithm Comparison

In the case of the departure operation scheduling of 12 shipboard aircraft, we have sorted out the following information in Table 8 based on the solution results of three algorithms. Furthermore, we conducted pairwise *t*-tests between the LTA-HPSO + AAE-SAC algorithm and the PSO + heuristic rule algorithm, as well as the PSO + SAC algorithm. The statistical results show that in the one-sided *t*-test with the PSO + heuristic rule algorithm, the statistical result is $t\left(9\right)=3.515>1.833,\;p=0.007<0.05$, and the corresponding *p*-value is much smaller than the null hypothesis, indicating a significant difference in the optimization performance of the two algorithms. In the one-sided *t*-test with the PSO + SAC algorithm, the statistical result is $t\left(18\right)=5.73>1.734,\;p=0.00028<0.05$, and the corresponding *p*-value is much smaller than the null hypothesis, indicating a significant difference in the optimization performance of the two algorithms. Meanwhile, the average time taken by the LTA-HPSO + AAE-SAC algorithm to obtain the optimal scheduling is 652.6 s, with an average running time of approximately 125 s. Compared with the PSO + SAC algorithm under the same framework, the average scheduling time is shortened by 13.75%, and the operation time is reduced by 30.48%. Although compared with the traditional PSO + heuristic rule, in solving small-scale scheduling, the operation time cost does not show an advantage, the average scheduling time of the optimal solution of the LTA-HPSO + AAE-SAC algorithm is shortened by 31.27%.

In the large-scale scheduling case of 24 shipboard aircrafts taking off and leaving the field, the solution results of the three algorithms are summarized in Table 9. A *t*-test was conducted for pairwise comparisons between the LTA-HPSO + AAE-SAC algorithm and the PSO + heuristic rule algorithm, as well as the PSO + SAC algorithm. The statistical results show that in the one-sided *t*-test with the PSO + heuristic rule algorithm, $t\left(9\right)=8.445>1.833,\;p=0.00001<0.05$, which is significantly smaller than the null hypothesis, indicating a significant difference in the optimization performance of the two algorithms. In the one-sided *t*-test with the PSO + SAC algorithm, $t\left(9\right)=9.918>1.833,\;p=0.00000<0.05$, which is significantly smaller than the null hypothesis, indicating a significant difference in the optimization performance of the two algorithms. Moreover, all *t*-test results are far smaller than the null hypothesis, fully demonstrating that the algorithm proposed in this paper has a more prominent optimization capability in large-scale scheduling problems. Although the scheduling of 24 aircraft involves different priorities, hangar scheduling, and deck support, which increases the complexity of the takeoff and departure operations, the average scheduling time obtained by the LTA-HPSO + AAE-SAC algorithm is still 49.54% shorter than that of the PSO + heuristic rule algorithm and 26.81% shorter than that of the PSO + SAC algorithm. At the same time, the average running time of the algorithm has only increased by 48.04% compared to itself, and compared to the two benchmark algorithms, it shows improved superiority and good solution stability.

#### 4.3.2. Sensitivity Analysis

**Figure 17:**
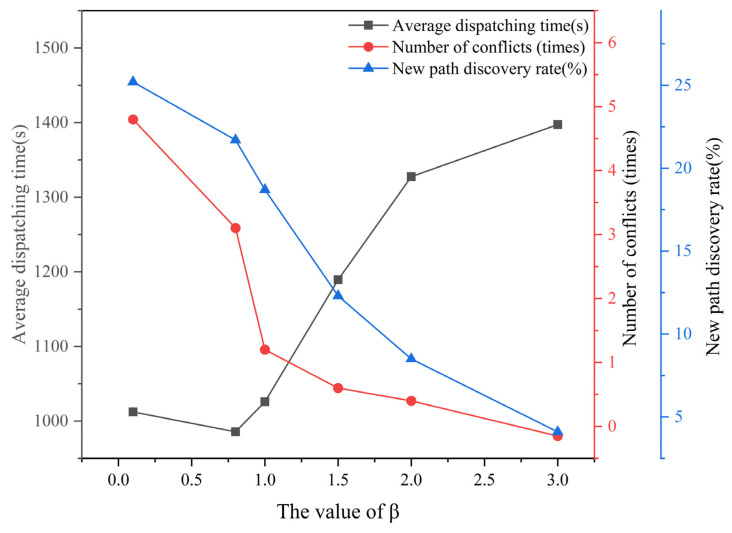
The influence of the value of the dynamic target entropy parameter β in the AAE-SAC algorithm on the performance of the two-stage scheduling optimization algorithm.

In the preceding section, we demonstrated the superiority of the LTA-HPSO + AAE-SAC algorithm by comparing it with two other benchmark algorithms. In this section, we provide a detailed analysis to verify the rationality and feasibility of the improvements made to it. Specifically, to enhance the coordination capability and new-path exploration of the AAE-SAC algorithm for shipboard aircraft deck scheduling via the dynamic target entropy adjustment mechanism (DTEAM), we performed a sensitivity analysis on the parameter β in Equation (44). Using a scenario with 24 aircraft departures, we conducted 100 independent simulation experiments. The β value range was set to (0, 3]. We examined the effects of varying β values on aircraft departure operation time and the relationships between β and the occurrences of conflicts and path exploration during cooperative scheduling. Figure 17 presents a statistical chart illustrating the relationships among the dynamic target entropy parameter β, scheduling time, number of conflicts, and proportion of new paths. Based on the results shown in the figure, we summarize the following observations: as the β value increases, the scheduling time for shipboard aircraft departures (black line) increases, the number of conflicts, such as aircraft collisions, wake interference, and position occupation (red line), decreases, and the proportion of new paths (blue line) employed decreases. Consequently, we conclude that when the parameter *β* falls within the range [0.8, 1.5], the AAE-SAC algorithm achieves effective performance in collaborative path planning for large-scale aircraft departure operations and optimally balances conflict avoidance and path exploration during the scheduling process.

**Figure 18:**
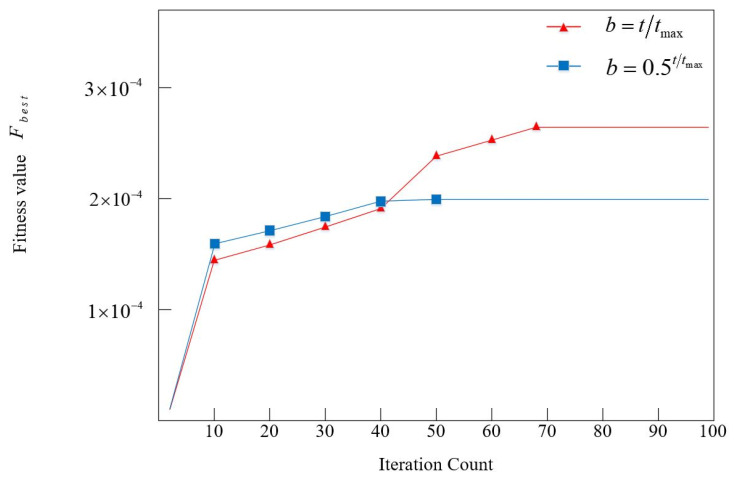
Performance analysis of the impact of scale parameter b in Laplace perturbation on the fitness of LTA-HPSO algorithm.

In the enhancement of the Particle Swarm Optimization (PSO) algorithm, a Laplace perturbation is incorporated into the original single-learning factor, enabling this parameter to vary dynamically throughout the iterative procedure. This modification enhances the algorithm’s optimization capability within the particle solution space. For the scale parameter b of the Laplace perturbation function, the linear form (Equation (61)) is adopted. To validate the design rationale, a comparative experiment was conducted involving 30 independent simulation trials, contrasting the linear form with an exponential form defined as $b=0.5^{t/t_{\max}}$. Using a case study of 24-aircraft scheduling with 100 generations per trial, the statistical mean distribution is shown in Figure 18. The blue trajectory illustrates the impact of the exponential-form Laplace scale parameter on the algorithm’s fitness value, which fully converges by generation 50. In contrast, the red trajectory depicting the linear-form’s influence converges at generation 68 but achieves a significantly higher optimal fitness value than the exponential counterpart. These results demonstrate that the linear-form Laplace perturbation facilitates the LTA-HPSO algorithm in attaining superior minimum scheduling time solutions for aircraft departure scheduling optimization.

## 5. Conclusions

This paper investigates the scheduling optimization problem in deck departure operations of shipboard aircraft. We propose a novel two-stage optimization algorithm (LTA-HPSO + AAE-SAC) to address this problem. Building upon the particle swarm optimization (PSO) algorithm, we introduce a population segmentation strategy based on task sequence and take-off sequence. By incorporating Laplacian perturbation learning factors and a dynamic tabu table, we develop the enhanced LTA-HPSO algorithm, significantly improving optimization quality and efficiency. Concurrently, the AAE-SAC algorithm is proposed by dynamically adjusting policy entropy in the SAC algorithm and integrating an attention mechanism. This enhancement effectively mitigates the limitations in collaborative trajectory optimization for large-scale aircraft departure operations. The integration of PSO and deep reinforcement learning (DRL) within a two-stage framework represents a novel approach in carrier-based departure operations research, resolving the key limitation of existing studies which treated scheduling schemes and spatial paths in isolation.

Case simulation results demonstrate that the LTA-HPSO + AAE-SAC algorithm effectively tackles departure scheduling problem across different scales, outperforming two benchmark algorithms in both solution quality and stability. Although the traditional approach (PSO + heuristic rules) offers faster solution times for small-scale problems, LTA-HPSO + AAE-SAC incurs computational overhead due to sample training and real-time path control. However, this investment resolves the inherent imbalance in traditional two-stage scheduling, characterized by low coherence between scheduling schemes and their corresponding paths, as well as path discontinuity. The average scheduling time achieved by the optimal solution using LTA-HPSO + AAE-SAC is reduced by 31.27% compared to PSO + heuristic rules and by 13.75% compared to PSO + SAC. For a complex scenario involving 24 aircraft with varying priorities and heterogeneous tasks, the algorithm enables real-time collaborative path planning on the deck, with only a 48.04% increase in computational time. Notably, it enhances multi-aircraft operational safety while demonstrating superior robustness for complex scheduling scenarios.

Therefore, this study provides an effective solution for scheduling optimization within the trend of large-scale, high-dynamic, and highly collaborative aircraft departure operations, thereby enriching theoretical frameworks for deck operations and enhancing aircraft departure efficiency. Furthermore, the methodological framework holds applicability potential for scheduling problems requiring integrated path consideration, such as workshop scheduling.

The dynamic aspects considered in this study pertain primarily to deck space and priority changes. However, we did not account for uncertainties involved in hardware operations, such as aircraft warm-up time and deflector plate cooling time. Additionally, the impact of support personnel on operation times and the scheduling challenges in mixed manned/unmanned scenarios require further investigation. While the AAE-SAC algorithm utilizes an attention mechanism to reduce the dimensionality of deck state space, exploring its limitations under increased complexity involving more aircraft, personnel states, and surrounding factors will be a key focus of future research.

## Figures and Tables

**Table 1:** Priority relationships in carrier-based aircraft departure operation.

Type of Aircraft	Priority Rank	$\mathrm{Value}\;\mathrm{of}\;{\mathrm{Pr}}_{Ci}$	Type of Mission	Priority Rank	$\mathrm{Value}\;\mathrm{of}\;{\mathrm{Pr}}_{Mi}$
Early-warning aircraft	I	3	Ground strike	I	3
Heavy aircraft	II	2	Air-to-air	II	2
Stealth/electronic aircraft	III	1	Cruise	III	1

**Table 2:** Parameters of the LTA-HPSO algorithm.

Parameter Name	Value
Population size	100
Maximum number of iterations	100
$\mathrm{Base}\;\mathrm{individual}\;\mathrm{learning}\;\mathrm{factor}\;c_{1}$	0.5
$\mathrm{Base}\;\mathrm{social}\;\mathrm{learning}\;\mathrm{factor}\;c_{2}$	0.5
$\mathrm{Inertia}\;\mathrm{weight}\;\omega_{\max}$	1.2
$\mathrm{Inertia}\;\mathrm{weight}\;\omega_{\min}$	0.15

**Table 3:** Aircraft departure operation-related parameters of the simulation system.

Parameter Name	Values and Units	Parameter Name	Values and Units
Landing Gear Retention Time for Aircraft	40 s	Preparation Time for Bend Plate	30 s
Release Time for Landing Gear of Aircraft	30 s	Cooling and Reset Time for Bend Plate	30 s
Maximum Traction Speed	1.5 m/s	Pre-Takeoff Inspection Time	30 s
Maximum Taxiing Speed	3.0 m/s	Interval Time of take-off	30 s
Transport Time for Lifting Gear	60 s	Engine Warm-Up Time	60 s
One-stop Support Time	8 min		

**Table 4:** Results of Case 1 where 10 simulations of aircraft departure operations are solved by three algorithms.

Number of Algorithm Executions	PSO + Heuristic		PSO + SAC		LTA-HPSO + AAE–SAC	
	Optimal Solution Scheduling Time/s	Algorithm Running Time/s	Optimal Solution Scheduling Time/s	Algorithm Running Time/s	Optimal Solution Scheduling Time/s	Algorithm Running Time/s
Ex1	841	38.64	709	157.64	692	136.56
Ex2	870	79.87	734	150.87	635	115.85
Ex3	848	68.65	706	119.84	699	134.05
Ex4	815	15.36	768	200.63	661	128.72
Ex5	864	64.62	811	271.58	716	143.21
Ex6	844	34.35	738	162.23	625	108.46
Ex7	1663	129.25	835	256.83	653	140.84
Ex8	868	69.35	792	223.59	622	126.23
Ex9	1066	96.62	710	122.68	609	102.63
Ex10	816	21.44	763	133.65	614	114.42

**Table 5:** Scheduling plan of Case 1.

Aircraft Number	Priority	Parking Position Number	Take-Off Position Number	Take-Off Sequence
1	3	A2	C3	1
2	3	A1	C3	4
3	3	B8	C2	6
4	3	B7	C1	5
5	3	B13	C1	2
6	3	B14	C3	7
7	3	A9	C2	3
8	3	A10	C1	8
9	3	A11	C2	9
10	3	A12	C3	10
11	3	B15	C1	11
12	3	B16	C2	12

**Table 6:** Results of Case 2 in which 10 simulations of aircraft departure operations are solved by three algorithms.

Number of Algorithm Executions	PSO + Heuristic		PSO + SAC		LTA-HPSO + AAE–SAC	
	Optimal Solution Scheduling Time/s	Algorithm Running Time/s	Optimal Solution Scheduling Time/s	Algorithm Running Time/s	Optimal Solution Scheduling Time/s	Algorithm Running Time/s
Ex1	2013	635.187	1354	365.354	991	193.820
Ex2	2251	678.534	1314	354.581	985	201.801
Ex3	2595	751.684	1473	439.950	984	176.725
Ex4	1743	594.953	1311	355.315	1062	214.395
Ex5	1976	628.512	1387	412.790	995	191.963
Ex6	2639	819.926	1293	349.201	1010	205.662
Ex7	1640	583.541	1554	437.165	1076	186.946
Ex8	1797	581.664	1391	381.300	1093	234.839
Ex9	1951	679.224	1606	540.937	988	166.274
Ex10	1874	597.821	1332	414.956	1074	189.460

**Table 7:** Scheduling plan for Case 2.

Aircraft Number	Priority	Parking Position Number	Take-Off Position Number	Take-Off Sequence
1	5	A4	C3	1
2	5	A3	C2	2
3	3	A2	C1	3
4	3	A1	C3	4
5	3	A5	C3	7
6	3	A6	C2	8
7	3	A7	C1	9
8	3	A8	C3	10
9	3	A9	C1	15
10	4	B2	C2	5
11	4	B1	C1	6
12	3	A10	C2	14
13	3	A11	C1	12
14	3	A12	C2	11
15	3	A13	C3	13
16	3	A14	C3	16
17	3	A15	C2	17
18	3	A16	C1	18
19	3	B8 → P1 → B13	C1	23
20	3	B7 → P2 → B14	C2	24
21	3	B13 → P3 → A9	C3	19
22	3	B14 → P4 → A10	C1	20
23	3	B15 → P5 → A11	C2	21
24	3	B16 → P6 → A12	C3	22

**Table 8:** Statistical analysis results of aircraft departure operations obtained by three algorithms in Case 1.

Algorithm Name	Optimal Scheduling Time (s)	Average Scheduling Time (±Standard Deviation)	Average Algorithm Running Time (s)
PSO + heuristic	815	949.50 ± 260.78	61.81 ± 35.39
PSO + SAC	706	756.60 ± 45.19	179.95 ± 55.10
LTA-HPSO + AAE–SAC	609	652.60 ± 38.28	125.09 ± 14.07

**Table 9:** Statistical analysis results of aircraft departure operation obtained by three algorithms in Case 2.

Algorithm Name	Optimal Scheduling Time (s)	Average Scheduling Time (±Standard Deviation)	Average Algorithm Running Time (s)
PSO + heuristic	1640	2032.90 ± 354.45	655.10 ± 78.98
PSO + SAC	1293	1401.50 ± 108.12	405.15 ± 58.83
LTA-HPSO + AAE–SAC	984	1025.80 ± 44.62	185.19 ± 17.18

## Data Availability

The original contributions presented in this study are included in the article. Further inquiries can be directed to the corresponding author.
